# IFNγ and IL-12 Restrict Th2 Responses during Helminth/*Plasmodium* Co-Infection and Promote IFNγ from Th2 Cells

**DOI:** 10.1371/journal.ppat.1004994

**Published:** 2015-07-06

**Authors:** Stephanie M. Coomes, Victoria S. Pelly, Yashaswini Kannan, Isobel S. Okoye, Stephanie Czieso, Lewis J. Entwistle, Jimena Perez-Lloret, Nikolay Nikolov, Alexandre J. Potocnik, Judit Biró, Jean Langhorne, Mark S. Wilson

**Affiliations:** 1 Division of Molecular Immunology, The Francis Crick Institute, London, United Kingdom; 2 Division of Systems Biology, The Francis Crick Institute, London, United Kingdom; 3 Division of Parasitology, Mill Hill Laboratories, London, United Kingdom; University of York, UNITED KINGDOM

## Abstract

Parasitic helminths establish chronic infections in mammalian hosts. Helminth/*Plasmodium* co-infections occur frequently in endemic areas. However, it is unclear whether *Plasmodium* infections compromise anti-helminth immunity, contributing to the chronicity of infection. Immunity to *Plasmodium* or helminths requires divergent CD4^+^ T cell-driven responses, dominated by IFNγ or IL-4, respectively. Recent literature has indicated that Th cells, including Th2 cells, have phenotypic plasticity with the ability to produce non-lineage associated cytokines. Whether such plasticity occurs during co-infection is unclear. In this study, we observed reduced anti-helminth Th2 cell responses and compromised anti-helminth immunity during *Heligmosomoides polygyrus* and *Plasmodium chabaudi* co-infection. Using newly established triple cytokine reporter mice (*Il4^gfp^Ifng^yfp^Il17a^FP635^*), we demonstrated that *Il4^gfp+^* Th2 cells purified from *in vitro* cultures or isolated *ex vivo* from helminth-infected mice up-regulated IFNγ following adoptive transfer into *Rag1^–/–^* mice infected with *P*. *chabaudi*. Functionally, Th2 cells that up-regulated IFNγ were transcriptionally re-wired and protected recipient mice from high parasitemia. Mechanistically, TCR stimulation and responsiveness to IL-12 and IFNγ, but not type I IFN, was required for optimal IFNγ production by Th2 cells. Finally, blockade of IL-12 and IFNγ during co-infection partially preserved anti-helminth Th2 responses. In summary, this study demonstrates that Th2 cells retain substantial plasticity with the ability to produce IFNγ during *Plasmodium* infection. Consequently, co-infection with *Plasmodium* spp. may contribute to the chronicity of helminth infection by reducing anti-helminth Th2 cells and converting them into IFNγ-secreting cells.

## Author Summary

Approximately a third of the world’s population is burdened with chronic intestinal parasitic helminth infections, causing significant morbidities. Identifying the factors that contribute to the chronicity of infection is therefore essential. Co-infection with other pathogens, which is extremely common in helminth endemic areas, may contribute to the chronicity of helminth infections. In this study, we used a mouse model to test whether the immune responses to an intestinal helminth were impaired following malaria co-infection. These two pathogens induce very different immune responses, which, until recently, were thought to be opposing and non-interchangeable. This study identified that the immune cells required for anti-helminth responses are capable of changing their phenotype and providing protection against malaria. By identifying and blocking the factors that drive this change in phenotype, we can preserve anti-helminth immune responses during co-infection. Our studies provide fresh insight into how immune responses are altered during helminth and malaria co-infection.

## Introduction

Infections with *Plasmodium* and helminths are extremely common, each contributing to substantial morbidity in affected populations [1–3]. Additionally, co-infections with *Plasmodium* species and intestinal helminths occur frequently in co-endemic areas [4,5]. The impact of co-infection on disease burden, pathogenesis, resistance to infection and immunity is complex and poorly understood. The vast majority of reported co-infection studies have focused on the impact of helminth infection on *Plasmodium*-associated responses, identifying altered anti-malarial immune responses or malaria-associated pathology during helminth co-infection [6–11]. However, the specific impact of *Plasmodium* infection on anti-helminth immunity has not been well characterized. Experimental murine models of helminth and *Plasmodium* co-infections have been established, however these have also mainly focused on how concomitant helminth infection affects *Plasmodium* immunity and pathology [11–16], with much less focus on how *Plasmodium* infection impacts helminth-associated type 2 responses.

Murine models of intestinal helminth infections have delineated a clear role for Th2-directed immune responses for proficient immunity. In particular, infection with the natural murine helminth, *Heligmosomoides polygyrus*, results in a chronic infection with the induction of a polarized type 2 response, characterized by IL-4-producing Th2 cells, alternative activation of macrophages and elevated IgE, closely mimicking human helminthiasis. Following anthelmintic treatment, Th2 cell-dependent immunity protects mice from re-infection (reviewed in [17,18]). In contrast, acute blood-stage infection with the rodent malaria parasite, *Plasmodium chabaudi chabaudi (*AS), results in polyclonal lymphocyte activation with a strongly polarized Th1 response [19]. Disease is associated with a spectrum of immunopathologies including splenomegaly and anemia [20–22] with peak parasitemia occurring 7–9 days post-infection [23]. These well-studied experimental systems, modeling human disease, provide appropriate tools to dissect the immune responses during co-infection.

There is a large body of literature describing the antagonistic relationship between Th1 and Th2 cell differentiation. *In vitro*-based studies have clearly established that under Th1 and Th2 polarizing conditions, differentiated cells become more fixed in their phenotype with increasing rounds of cell division, losing their ability to convert to alternative phenotypes [24,25]. Mechanistically, T-bet and GATA-3, transcription factors required to promote Th1 and Th2 differentiation, respectively, inhibit differentiation of the opposing phenotype [26,27]. Despite this clear antagonistic relationship, IL-4^+^IFNγ^+^ and T-bet^+^GATA-3^+^ Th cells are readily observed *in vivo* [28,29], and several studies have established that Th subsets retain flexibility in their ability to produce non-lineage-specific cytokines [30–32]. Indeed, recent studies challenging the fate-lineage dogma demonstrated that antigen-restricted TCR transgenic Th2 cells co-produced IFNγ and IL-4 following LCMV infection [33,34].

In light of these new data, it is possible that Th cell conversion occurs during co-infection, altering immunity to one or both pathogens or contributing to the chronicity of helminth infection.

In this study, we observed that *Plasmodium* and helminth co-infection led to a reduction of helminth-elicited *Il4* ^*gfp+*^ Th2 cells and compromised anti-helminth immunity. We hypothesized that helminth-elicited Th2 cells were being converted into IFNγ-secreting Th1 cells during *Plasmodium* co-infection, as pressure to control both pathogens was placed on the Th cell population. To test this hypothesis, we generated triple cytokine reporter mice to accurately purify and identify *Il4* ^*gfp*^, *Ifng* ^*yfp*^ and *Il17a* ^*FP635*^-expressing cells to determine whether Th2 cells had the ability to change their phenotype. We observed that *Il4*-expressing Th2 cells could readily produce IFNγ following adoptive transfer in *Rag* ^*–/–*^recipients, and these cells reduced severe parasitemia during acute *P*. *chabaudi* infection. Conversion of Th2 cells was dependent upon IL-12 and IFNγ-signaling, and blockade of these cytokines during co-infection preserved the Th2 response. Overall, this study provides fresh insight into the functional relationship between IFNγ- and IL-4-producing Th cells during co-infection and indicates that limiting acute Th1 responses may preserve Th2-mediated anti-helminth immunity.

## Results

### *Plasmodium* infection compromises Th2-dependent anti-helminth immunity

**Fig 1:**
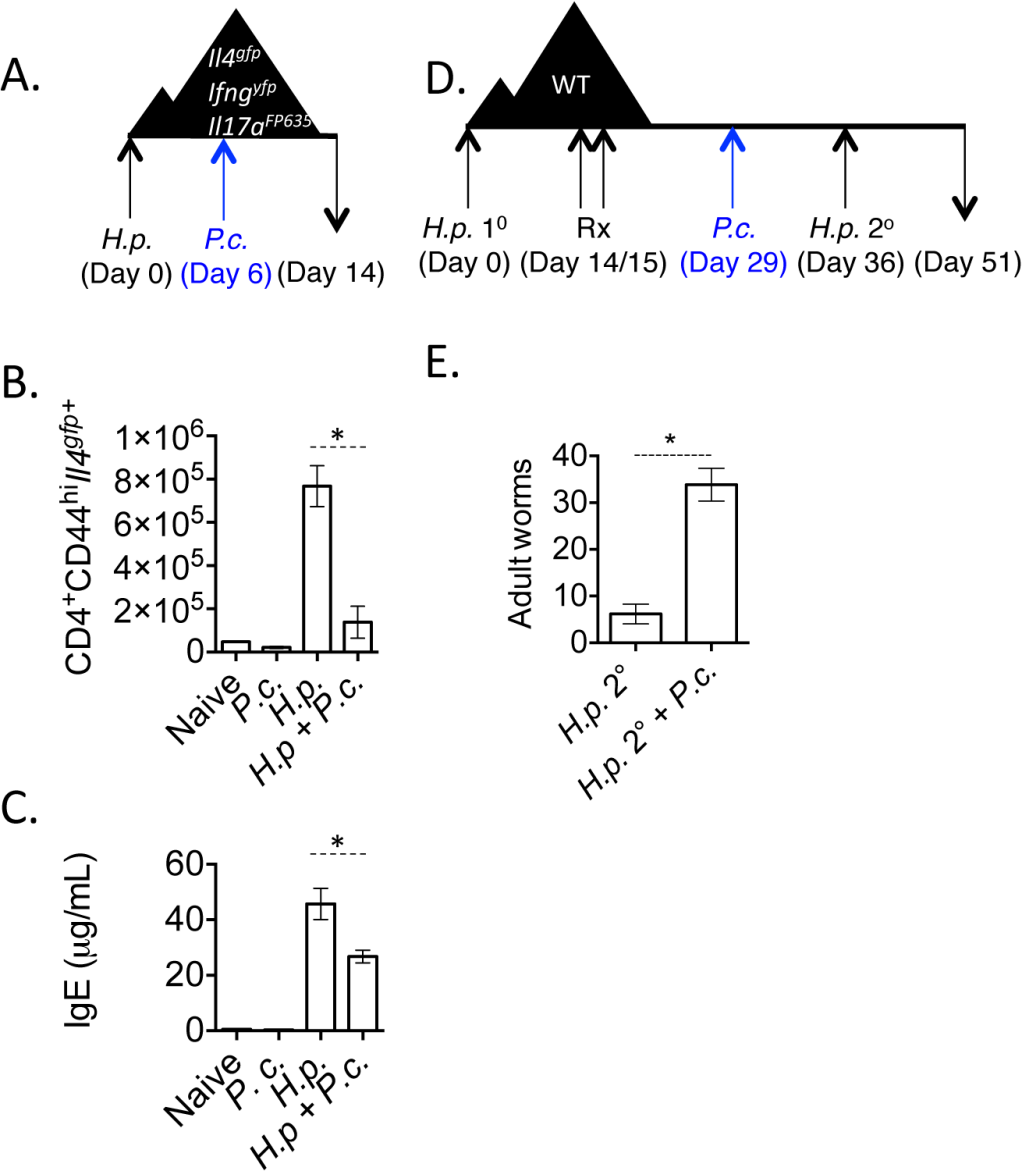
*H*. *polygyrus/ P*. *chabaudi* co-infection leads to impaired Th2 responses. A-C). Triple reporter mice were orally infected with 200 *H*. *polygyrus* larvae. 6 days post-infection, mice were infected i.p. with 10^5^ *P*. *chabaudi*. At day 8 of *P*. *chabaudi* infection (d14 *H*. *polygyrus*), mice were harvested. B). Total numbers of CD4^+^CD44^hi^ *Il4* ^gfp+^ cells in the mesenteric lymph nodes. Data are representative of 5 independent experiments with 2–4 mice per group. C). IgE measured in the serum by ELISA from 3 pooled experiments. D and E). C57BL/6 mice were infected with 200 *H*. *polygyrus* larvae, treated on 2 consecutive days (days 14–15) with pyrantel pamoate (5 mg), infected with 10^5^ *P*. *chabaudi*, and re-infected with *H*. *polygyrus*. Adult worms in intestine were counted on day 51. Data are representative of 4 independent experiments with 6–7 mice per group. * denotes P<0.05.

To assess the impact of concomitant *Plasmodium* infection on the development of Th2 responses, we infected mice with *H*. *polygyrus* and 6 days later with 10^5^ *P*. *chabaudi*-infected red blood cells (Fig 1A). To accurately identify simultaneous transcription of Th1 (*Ifng*), Th2 (*Il4)* and Th17 (*Il17a*) lineage-defining genes, we generated a triple cytokine reporter mouse (*Il4* ^*gfp*^ *Ifng* ^*yfp*^ *Il17a* ^*Cre*^ *R26* ^*FP635*^) using existing and new fluorescent cytokine reporter mouse strains [35–37] (S1 Fig). Following infection with L3 larvae of the intestinal helminth, *H*. *polygyrus*, we observed a significant expansion of *Il4* ^*gfp+*^ CD4^+^ Th2 cells in the mesenteric lymph nodes 14 days post-infection. Co-infected mice had significantly reduced numbers of *Il4* ^*gfp+*^ CD4^+^ Th2 cells in the mesenteric lymph nodes (Fig 1B) as well as a reduction in serum IgE (Fig 1C) and decreased expression of the alternative macrophage activation marker, *Retnla* (*Relmα*) in the gut (S2 Fig). These data indicated that helminth-elicited Th2 cells and Th2-driven immune responses were compromised during *Plasmodium* co-infection. The reduced *Il4* ^*gfp+*^ cells in the mesenteric lymph nodes correlated with an increase in *Ifng* ^*yfp+*^ cells in the spleen during co-infection.

Very few *Il17a* ^*FP635+*^ cells were induced in this model (S2 Fig). Following the resolution of acute malarial parasitemia, Th2 cell numbers in the mesenteric lymph nodes and serum IgE returned to levels observed in mice infected with *H*. *polygyrus* only (S2 Fig).

*H*. *polygyrus* establishes a chronic infection in wild type C57BL/6 mice. However, treating mice with anthelmintics kills adult parasites and allows a protective memory Th2 response to develop. Upon re-infection, mice expel worms in a CD4^+^ T cell- and IL-4-dependent manner [38,39]. Following the observation that *P*. *chabaudi* infection compromised Th2 cell responses (Fig 1B), we tested whether *P*. *chabaudi* infection would impact Th2-dependent anti-helminth immunity. We infected wild type mice with *H*. *polygyrus*, treated mice with the anthelmintic, pyrantel pamoate, and then infected mice with *P*. *chabaudi* 7-days prior to re-infection with *H*. *polygyrus* (Fig 1D). Although *H*. *polygyrus*-specific IgG1 levels were comparable between groups of mice (S2 Fig), *P*. *chabaudi*-infected mice that had been given a secondary *H*. *polygyrus* challenge infection had significantly more adult worms in the intestinal lumen (Fig 1E), indicating that *Plasmodium* infection compromised proficient anti-helminth immunity.

### *Il4* ^*gfp+*^ Th2 cells can functionally adapt, up-regulating IFNγ to control *Plasmodium* infection

**Fig 2:**
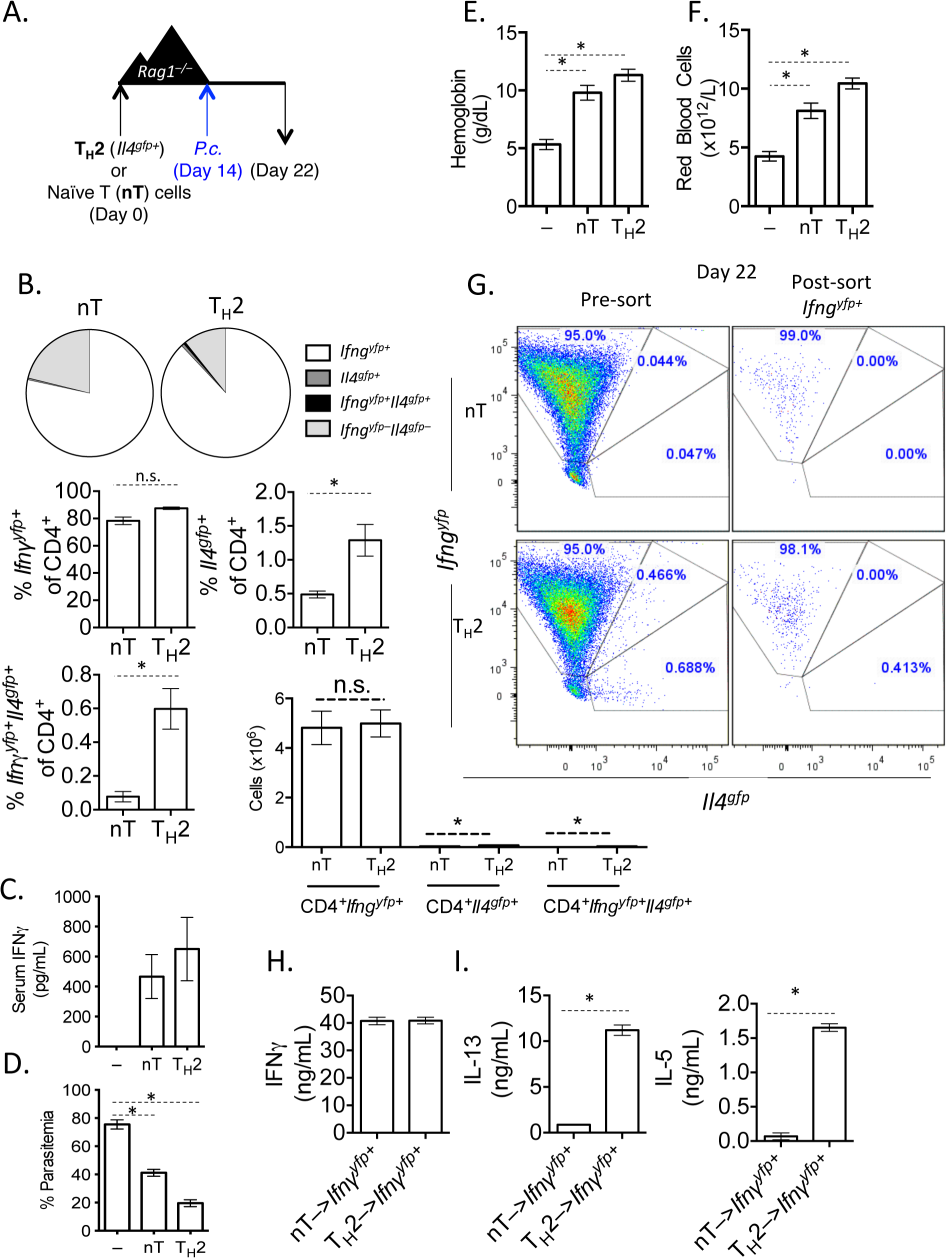
*In vitro* Th2 cells produce IFNγ and protect *Rag1* ^*–/–*^mice during *Plasmodium* infection. A). Experimental set-up: 2-week *in vitro* polarized Th2 cells were FACS sorted as CD4^+^ *Il4* ^*gfp+*^ *Ifng* ^*yfp–*^ *Il17a* ^*FP635–*^ and transferred i.v. to *Rag1* ^*–/–*^mice. As a control, a group of *Rag1* ^*–/–*^mice received naïve CD4^+^ T cells. A second control group received no T cells. Recipient mice were infected with 10^5^ *P*. *chabaudi* i.p. on day 14 post-transfer and harvested at day 8 post-infection. B). Percent and total number of CD4^+^ *Il4* ^*gfp+*^ and *Ifng* ^*yfp+*^ cells in the spleen, as determined by FACS. C). Serum IFNγ levels determined by ELISA. D). Percent parasitemia was determined by blinded counting of Giemsa-stained blood smears. E and F). Hemoglobin and eHred blood cell counts were measured in peripheral blood by Vetscan. Data is representative of at least 3 independent experiments, with 3–5 mice per group. G). Converted CD4^+^TCRβ^+^ *Ifng* ^*yfp+*^ *Il4* ^*gfp–*^ *Il17a* ^*FP635–*^ cells were sorted from pooled spleens of 3 recipient *Rag1* ^*–/–*^mice at day 8 post-*P*. *chabaudi* infection. H). Sorted *Ifng* ^*yfp+*^ cells were cultured *in vitro* in Th2 conditions for 5 days. ELISAs for IFNγ (H), IL-5 and IL-13 (I) were run on cell supernatants. Error bars represent technical replicates. Data are representative of 3 independent experiments. * denotes P<0.05.

It has become clear in recent years that lineage-committed CD4^+^ T cells retain a degree of plasticity, with the ability to convert between phenotypes [30]. *Plasmodium* infection elicits a polyclonal expansion of lymphocytes and IFNγ-secreting T cells [21,22]. We therefore hypothesized that the loss of *Il4* ^*gfp+*^ Th2 cells in the mesenteric lymph nodes and the increase in *Ifng* ^*yfp+*^ cells in the spleen during *H*. *polygyrus* and *P*. *chabaudi* co-infection was due to conversion of Th2 cells to an IFNγ-producing Th1-like phenotype. To test whether Th2 cells could produce IFNγ during *P*. *chabaudi* infection, we FACS-purified CD4^+^TCRβ^+^ *Il4* ^*gfp+*^ *Ifng* ^*yfp–*^ *Il17a* ^*FP365–*^ Th2 cells from 2-week *in vitro* cultures (S1 Fig), adoptively transferred them into *Rag1* ^*–/–*^mice and infected the recipient mice with *P*. *chabaudi*. Cytokine expression in the transferred cells was analyzed in the spleen at day 8 post-infection (Fig 2A). Transferred Th2 cells (*Il4* ^*gfp+*^ *Ifng* ^*yfp–*^ *Il17a* ^*FP365–*^) almost completely lost expression of *Il4* ^*gfp*^ and, comparable to naïve T cells, expanded with approximately 80% of cells expressing *Ifng* ^*yfp*^ (Fig 2B). *Il17a* ^*FP635*+^ cells were barely detectable (<1%) following *Plasmodium* infection, in line with previous data [21,22,40]. IFNγ protein was also detectable in the serum of mice that received either naive CD4^+^ T cells or purified Th2 cells, but not in *P*. *chabaudi*-infected *Rag1* ^*–/–*^mice that received no T cells, indicating that serum IFNγ was T cell-dependent (Fig 2C). Thin blood smears from recipient mice identified that following infection of *Rag1* ^*–/–*^mice, very high parasitemia is observed (Fig 2D). The adoptive transfer of naïve T cells to *Rag1* ^*–/–*^mice significantly reduced the high parasitemia, confirming an important T cell-dependent role in the control of high parasitemia during acute infection. This system permitted us to test whether Th2 cells, which had converted into IFNγ^+^ cells, could also control high parasitemia following acute infection. Indeed, adoptive transfer of Th2 cells also significantly reduced parasitemia (Fig 2D), suggesting a functional loss of hemoglobin and severe anemia were also prevented in *Rag1* ^*–/–*^mice given Th2 cells (Fig 2E and 2F). Although Th2 cells up-regulated IFNγ in uninfected recipient *Rag1* ^*–/–*^mice, significantly greater expansion of these converted cells occurred in *P*. *chabaudi* infected recipient mice (S3 Fig). These data demonstrate that purified *Il4*-expressing Th2 cells were capable of producing IFNγ and could protect mice during acute *P*. *chabaudi* infection, similar to naive CD4^+^ T cells. Finally, to determine whether Th2 cells had the capacity to produce non-lineage cytokines in another model system, we infected *Rag1* ^*–/–*^recipient mice with *Candida albicans* (S4 Fig). At day 6 post *C*. *albicans* infection, transferred *Il4* ^*gfp+*^ Th2 cells had lost *Il4* expression and up-regulated IFNγ, similar to *P*. *chabaudi* infection. Interestingly, transferred Th2 cells did not up-regulate IL-17a, unlike naïve controls (S4 Fig).

### Th2 cells that have down-regulated *Il4* and up-regulated *Ifng* undergo significant transcriptional re-wiring yet retain the ability to produce IL-5 and IL-13

We next asked whether Th2 cells that had down-regulated *Il4* ^*gfp*^ and expressed *Ifng* ^*yfp*^ retained the ability to re-express Th2-associated cytokines. We transferred *Il4* ^*gfp+*^ Th2 cells into *Rag1* ^*–/–*^mice and infected recipient mice with *P*. *chabaudi*, as in Fig 2A. At day 8 post-infection with *P*. *chabaudi*, we sorted CD4^+^TCRβ^+^ *Ifng* ^*yfp+*^ *Il4* ^*gfp-*^ *Il17a* ^*FP635–*^ cells from the spleens of recipient mice (Fig 2G). Converted cells were then cultured *in vitro* with IL-4 and TCR stimulation. As expected, *Ifng* ^*yfp+*^ cells that were previously either naïve or *Il4* ^*gfp+*^ secreted IFNγ protein (Fig 2H), validating the fidelity of the transcriptional reporter system. However, only *Ifng* ^*yfp+*^ cells that were previously *Il4* ^*gfp+*^ secreted the Th2-associated cytokines IL-13 and IL-5 (Fig 2I), indicating that converted cells were indeed plastic, retaining the ability to produce Th2 cytokines.

**Fig 3:**
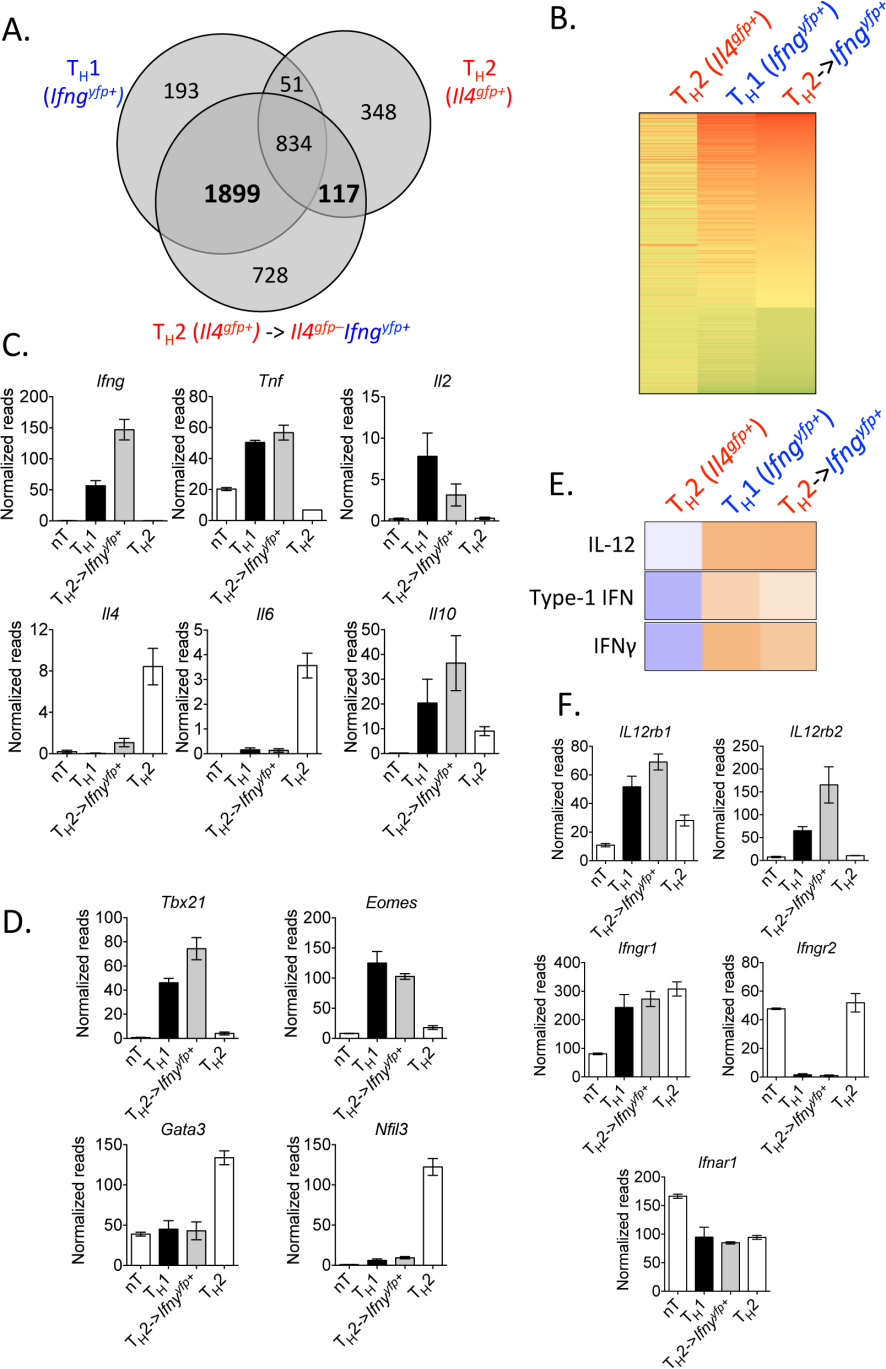
Converted Th2 cells are transcriptionally similar to Th1 cells. Purified *in vitro* Th2 cells (CD4^+^TCRβ^+^ *Il4* ^*gfp+*^ *Ifng* ^*yfp-*^ *Il17a* ^*FP635-*^) or naive CD4^+^ T cells were sorted for RNA or transferred to *Rag1* ^*–/–*^mice. Recipients were then infected with 10^5^ *P*. *chabaudi*, as in Fig 2. CD4^+^TCRβ^+^ *Ifng* ^*yfp+*^ *Il4* ^*gfp-*^ *Il17a* ^*FP635–*^ cells were then sorted from spleens of recipient mice at day 8 post-infection for RNA. RNA sequencing and IPA analysis was performed on the four cell populations. Data were expressed relative to naïve in A and B. A and B). Venn diagram and heatmap generated from differentially regulated genes (P<0.05, 2-fold relative to naïve T cells) of Th2 cells and Th1 cells, highlighting 1899 genes commonly expressed, which were not changed in Th2 cells. Th2 cells and converted Th2 cells shared 117 differentially regulated genes, which were not expressed in Th1 cells. C, D, and F). Normalized RNA-Seq reads of indicated genes. E). Upstream pathways analysis in Ingenuity Pathways Analysis (IPA) identified IL-12, type 1 IFN, and IFNγ as potential upstream regulators of converted Th2 cells. Samples were generated from 3 biological replicates (each sample representing cells from a single donor mouse).

To identify the degree of transcriptional re-wiring of the converted cells in this model, we performed RNA sequencing on Th2 cells (*Il4* ^*gfp+*^), converted Th2 cells (*Il4* ^*gfp+*^ → *Ifng* ^*yfp+*^ *Il4* ^*gfp-*^ *)*, naïve CD4^+^ T cells, and Th1 cells (naïve → *Ifng* ^*yfp+*^ *Il4* ^*gfp–*^ *)*, using the same sorting strategy as in Fig 2G. Comparing the transcriptome of all significantly differentially regulated genes (p<0.05, >2-fold relative to naive T cells) between the populations, we identified that converted cells had adopted a transcriptional profile very similar to Th1 cells (Fig 3A and 3B, S1 Table) with the majority of differentially regulated genes common with Th1 cells, while retaining some transcriptional similarity with their Th2 origin. Converted cells expressed *Ifng*, *Tnf*, *Il2* and *Il10* and largely lost expression of *Il4* and *Il6*, in comparison to the Th2 controls (Fig 3C). Similarly, the transcriptional machinery in converted cells resembled Th1 cells with elevated *Tbx21* (Tbet) and *Eomes* and low expression of Th2-associated transcription factors *Gata3* and *Nfil3* (Fig 3D). To identify putative mechanistic pathways responsible for Th2 cell conversion, we used an upstream pathways algorithm to predict factors that may contribute to the observed transcriptional profile (Ingenuity Pathways Analysis). This analysis identified canonical Th1 differentiation factors including IL-12, IFNγ and type 1 IFN as potential upstream factors contributing to the observed transcriptional profile in converted cells (Fig 3E). Furthermore, converted cells expressed *Il12rb1*, *Il12rb2*, *Ifngr1* and *Ifnar1* (Fig 3F). In summary, converted Th2 cells had undergone significant re-wiring, closely resembling Th1 cells.

### IFNγ production by Th2 cells does not depend on lymphopenia and requires TCR engagement

**Fig 4:**
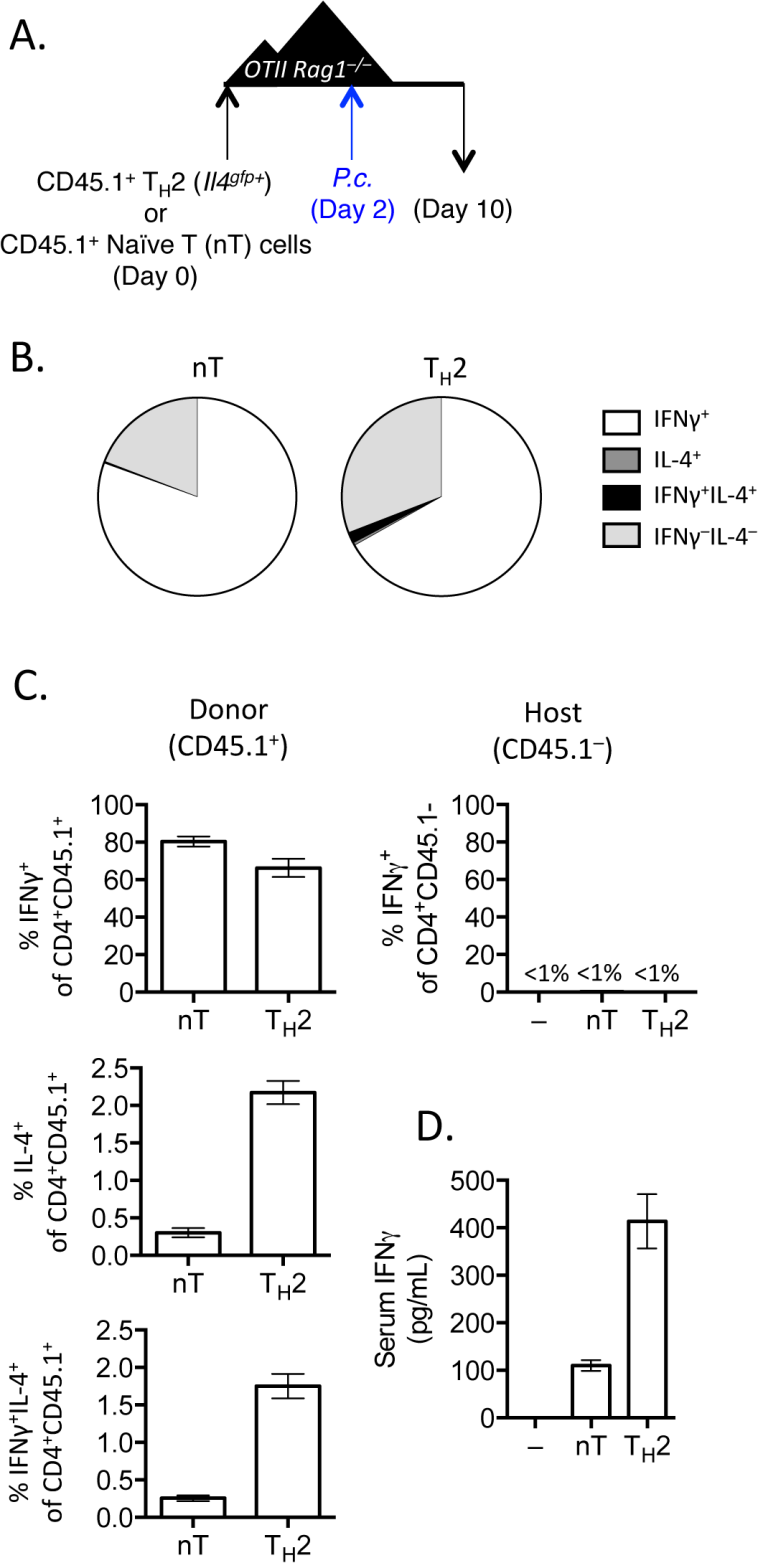
IFNγ production by Th2 cells does not depend on lymphopenia. A). Th2 cells generated from *Il4* ^*gfp*^ mice were polarized *in vitro* for 2 weeks, sorted as CD4^+^TCRβ^+^ *Il4* ^gfp+^, and 2.5x10^6^ were transferred to OTII *Rag1* ^*–/–*^recipient mice. Control groups received no T cells or sorted naïve T cells. 2 days post-transfer, mice were infected with 10^5^ *P*. *chabaudi*. B and C). Cytokine production in donor CD45.1^+^ or host cells in spleens, day 8 post-infection with *P*. *Chabaudi*, as determined by intracellular cytokine staining. D). IFNγ protein in serum, measured by ELISA. Data are representative of 2 independent experiments with 3–4 mice per group.

When T cells undergo expansion in lymphopenic environments a population of rapidly dividing cells up-regulate CD44 and IFNγ [41–43]. To test whether conversion of Th2 cells into IFNγ-expressing cells could occur in a CD4^+^ T cell replete mouse, we transferred purified Th2 cells or naïve CD4^+^ T cells into OTII *Rag1* ^*–/–*^mice [44], which have CD4^+^ T cells specific only for OVA peptide. We infected recipient mice with *P*. *chabaudi* and analyzed donor and host cells at day 8 post-infection (Fig 4A). Purified Th2 cells transferred into CD4^+^ OTII *Rag1* ^*–/–*^mice, similar to Th2 cells transferred into *Rag1* ^*–/–*^mice, produced IFNγ and down-regulated IL-4 (Fig 4B and 4C), contributing to elevated levels of serum IFNγ (Fig 4D). In contrast, host OVA-specific CD4^+^ T cells did not produce IFNγ following *Plasmodium* infection (Fig 4C). Thus, Th2 cell conversion was not dependent on lymphopenia.

**Fig 5:**
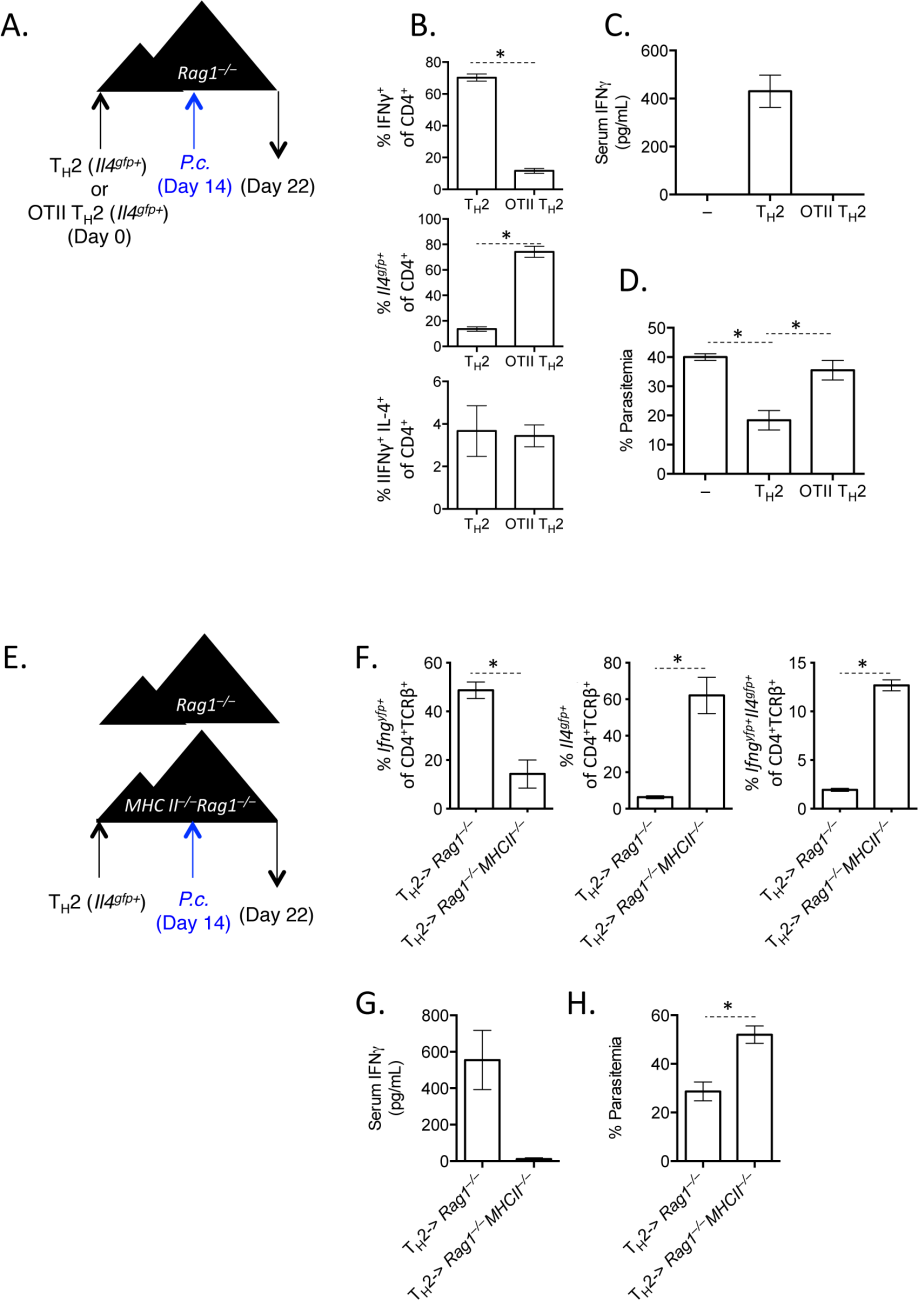
TCR stimulation is critical for IFNγ production by Th2 cells. A–D). OTII *Rag1* ^*–/–*^ *Il4* ^*gfp*^ or *Il4* ^*gfp*^ Th2 cells (CD4^+^TCRβ^+^ *Il4* ^*gfp+*^) were transferred to *Rag1* ^*–/–*^recipient mice. 14 days later, mice were infected with 10^5^ *P*. *chabaudi*, and mice were harvested at d8 post-infection. Representative of 2 independent experiments with 5 mice per group. B). Cytokine expression in transferred cells in the spleen (IFNγ by ICS, *Il4* ^*gfp*^ reporter expression). C). IFNγ protein in serum, measured by ELISA. D). Percent parasitemia, determined by blinded counting of Giemsa-stained blood smears. E–H). Th2 cells (CD4^+^TCRβ^+^ *Il4* ^*gfp+*^ *Ifng* ^*yfp-*^ *Il17a* ^*FP635-*^) were transferred to MHC Class II sufficient or deficient *Rag1* ^*–/–*^recipients. Mice were infected with 10^5^ *P*. *chabaudi* at day 14, and mice were harvested at day 8 post-infection. Representative of 2 independent experiments with 5 mice per group. F). Cytokine reporter expression in transferred cells in the spleen. G). IFNγ protein in serum, measured by ELISA. H). Percent parasitemia, determined by blinded counting of Giemsa-stained blood smears. * denotes P<0.05.

Given that Th cells require both TCR stimulation and cytokine-mediated signaling for differentiation, it was conceivable that pre-activated Th2 cells in this system would only require a second cytokine receptor-mediated signal to up-regulate IFNγ, without the need for any additional TCR stimulation. We took two independent approaches to test whether TCR engagement was required for Th2 cells to produce IFNγ. First, we generated and FACS-purified TCR-restricted Th2 cells from OTII *Rag1* ^*–/–*^mice crossed with *Il4* ^*gfp*^ reporter mice. We then transferred these OVA-specific *Il4* ^*gfp+*^ Th2 cells into *Rag1* ^*–/–*^recipients (devoid of OVA) and infected recipient mice with *P*. *chabaudi* (Fig 5A). Unlike polyclonal *Il4* ^*gfp+*^ Th2 cells that lost expression of *Il4* ^*gfp*^ and produced IFNγ, antigen-restricted OTII *Il4* ^*gfp+*^ Th2 cells retained expression of *Il4* ^*gfp*^ and failed to produce IFNγ (Fig 5B). Furthermore, IFNγ was not detectable in the serum of mice that received OVA-specific *Il4* ^*gfp+*^ Th2 cells (Fig 5C). Functionally, the failure to produce IFNγ correlated with significantly higher parasitemia, comparable to mice that received no T cells (Fig 5D). These data indicate that TCR signaling was required for the functional conversion of Th2 cells into IFNγ-secreting cells. To verify the requirement of TCR-signaling for conversion, we transferred purified *Il4* ^*gfp+*^ *Ifng* ^*yfp–*^ *Il17a* ^*FP365–*^ Th2 cells into *Rag1* ^*–/–*^recipient mice which were also deficient in MHC Class II and therefore unable to present antigens to *Il4* ^*gfp+*^ Th2 cells. Recipient mice were infected with *P*. *chabaudi*, and transferred cells were analyzed at day 8 post-infection (Fig 5E). As before, *Il4* ^*gfp+*^ Th2 cells transferred into MHC Class II-sufficient *Rag1* ^*–/–*^recipient mice down-regulated *Il4* ^*gfp*^ and up-regulated *Ifng* ^*yfp*^. However, *Il4* ^*gfp+*^ Th2 cells transferred to MHC Class II-deficient *Rag1* ^*–/–*^recipient mice remained *Il4* ^*gfp+*^, did not express *Ifng* ^*yfp*^ (Fig 5F) and failed to reduce severe parasitemia (Fig 5H). IFNγ was also undetectable in the serum (Fig 5G). Taken together, these two experimental systems demonstrate that conversion of Th2 cells in this model requires TCR engagement.

### IL-12 and IFNγ, but not type I IFN, promote IFNγ expression by transferred Th2 cells

**Fig 6:**
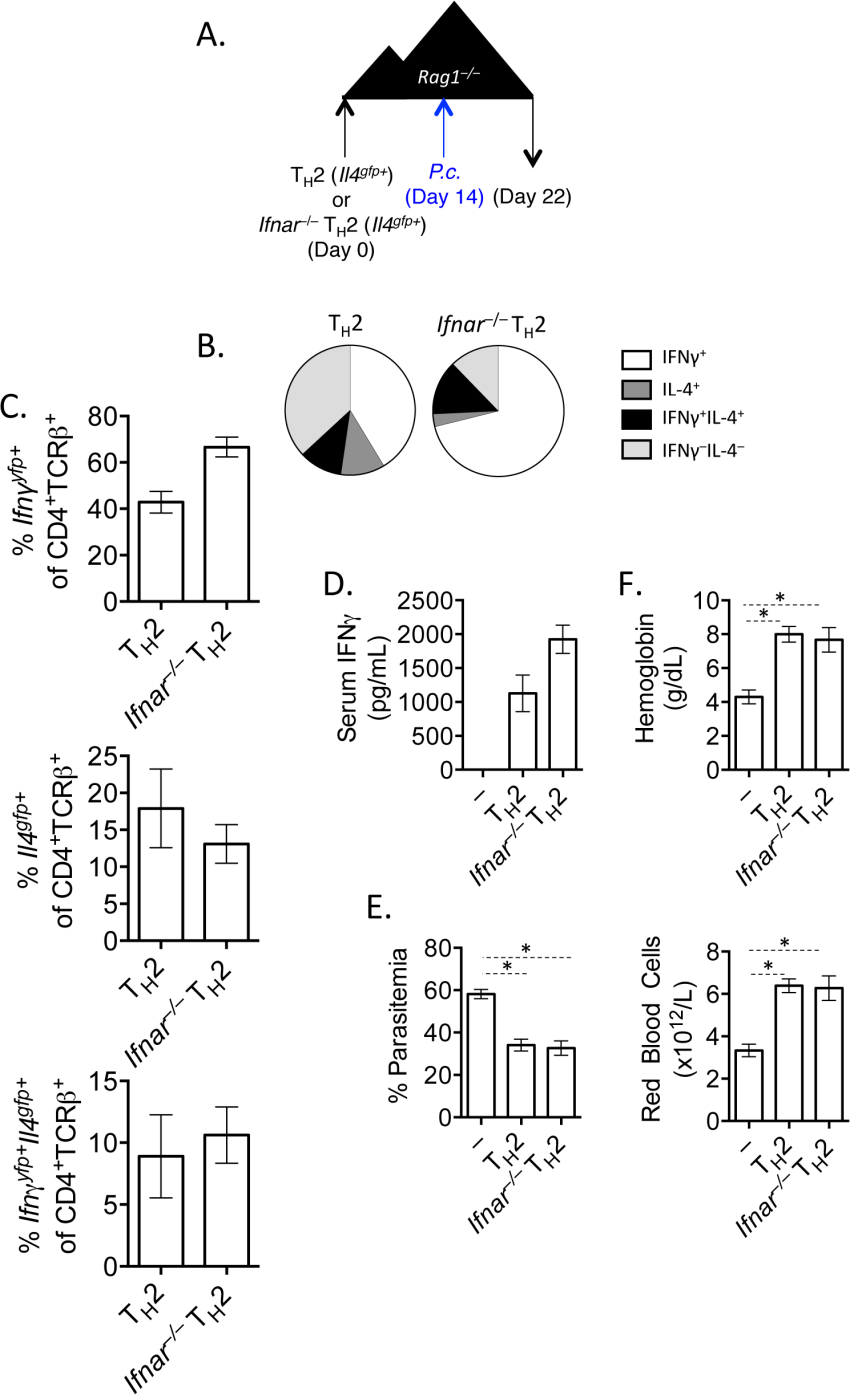
IFNγ production by Th2 cells does not depend of type I IFN. A). CD4^+^TCRβ^+^ *Il4* ^*gfp+*^ *Ifnar* ^*+/+*^ or *Ifnar* ^*–/–*^Th2 cells were transferred to *Rag1* ^*–/–*^mice. Recipient mice were infected with 10^5^ *P*. *chabaudi* 14 days later and mice were harvested at day 8 post-infection. B and C). Cytokine expression in transferred cells in the spleen (ICS). D). IFNγ protein in serum, measured by ELISA. E). Percent parasitemia, determined by blinded counting of Giemsa-stained blood smears. F). Hemoglobin and red blood cell counts determined by Vetscan. Data are representative of 2 independent experiments with 5–6 mice per group. * denotes P<0.05.

It has been shown previously that type I IFN signaling was required for IFNγ production from LCMV-specific TCR transgenic Th2 cells [34]. We had also observed that type 1 IFN was a candidate cytokine that could contribute to the transcriptional profile of converted Th2 cells (Fig 3E). We therefore tested the requirement for type 1 IFN signaling by crossing *Ifnar* ^*–/–*^mice with *Il4* ^*gfp*^ reporter mice. FACS purified *Il4* ^*gfp+*^ *Ifnar* ^*–/–*^ *or Il4* ^*gfp+*^ *Ifnar* ^*+/+*^ Th2 cells were transferred to *Rag1* ^*–/–*^recipient mice, subsequently infected with *P*. *chabaudi* and analyzed at day 8 post-infection (Fig 6A). Both type I IFN responsive and unresponsive Th2 cells were capable of up-regulating IFNγ (Fig 6B and 6C), contributing to serum IFNγ levels (Fig 6D). Furthermore, type I IFN responsive and unresponsive Th2 cells afforded similar protection from high parasitemia (Fig 6E), and prevented a loss in hemoglobin and red blood cells (Fig 6F). Thus, type I IFN signaling was dispensable for IFNγ production from ex-Th2 cells and for controlling high parasitemia.

**Fig 7:**
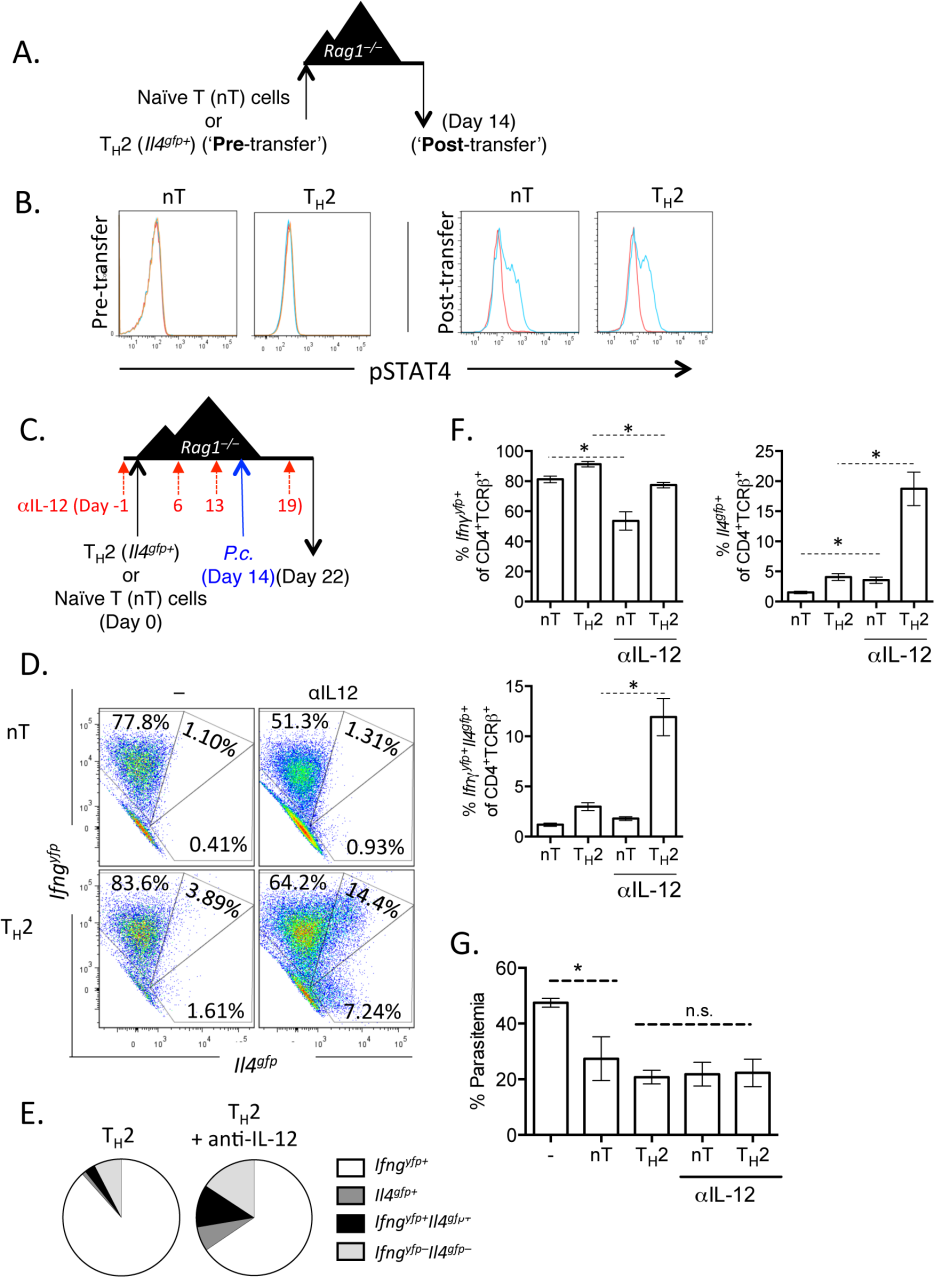
Th2 cells become IL-12 responsive following adoptive transfer. A and B). CD4^+^ *Il4* ^*gfp+*^ *in vitro* Th2 cells or naïve CD4 cells were transferred to *Rag1* ^*–/–*^mice for 2 weeks. CD4^+^TCRβ^+^ cells were then sorted from spleens of recipient mice and treated with 10ng/mL IL-12 for 15 minutes (blue) (or untreated, red) and then stained for pSTAT4 by FACS. Representative of 2 independent experiments. C—G). Naïve CD4^+^ T cells or *in vitro* Th2 cells (CD4^+^TCRβ^+^ *Il4* ^*gfp+*^ *Ifng* ^*yfp-*^ *Il17a* ^*FP635-*^) were transferred to *Rag1* ^*–/–*^recipient mice for 14 days. Mice were infected with *P*. *chabaudi* and harvested at day 8 post-infection. Mice were treated i.p. with 0.5mg of anti-IL12 at days -1, 6, 13, and 19. D–F). Cytokine reporter expression in transferred cells in the spleen, with or without anti-IL-12 treatment. G). Percent parasitemia, determined by blinded counting of Giemsa-stained blood smears. Data are representative of 2 independent experiments with 3–6 mice per group. * denotes P<0.05.

From our RNA-Seq analysis we also identified that the canonical Th1 differentiating cytokines, IL-12 and IFNγ, may be responsible for the transcriptional profile observed in our converted cells (Fig 3E). We first tested whether Th2 cells were responsive to IL-12 by measuring the phosphorylation of STAT4 following exposure to IL-12. Supporting previous studies [45–47], neither naïve CD4^+^ T cells nor sorted *Il4* ^*gfp+*^ Th2 cells phosphorylated STAT4 in response to IL-12 (Fig 7A and 7B; Pre- transfer). We then sorted transferred cells from naïve CD4^+^ T cell or *Il4* ^*gfp+*^ Th2 cell recipient *Rag1* ^*–/–*^mice 2 weeks post-transfer and found that both populations were responsive to IL-12 (Fig 7A and 7B; Post-transfer). Thus, it was possible that IL-12 was promoting IFNγ expression in Th2 cells following *P*. *chabaudi* infection. We tested the role of IL-12 by transferring naïve or *Il4* ^*gfp+*^ Th2 cells to *Rag1* ^*–/–*^mice and blocking IL-12 prior to and after *P*. *chabaudi* infection (Fig 7C). Blocking IL-12 reduced expression of *Ifng* ^*yfp*^ in naïve T cells (reduced from 78.9% to 52.61%); however, IL-12 blockade did not substantially alter the frequency of *Ifng* ^*yfp+*^ cells derived from Th2 cells. Instead, IL-12 blockade maintained expression of *Il4* ^*gfp+*^ in the Th2 population, with significantly larger *Il4* ^*gfp+*^ and *Il4* ^*gfp+*^ *Ifng* ^*yfp+*^ populations (Fig 7D–7F). These data indicate that in this system IL-12 down-regulated *Il4* ^*gfp*^ expression, but was not required for IFNγ from Th2 cells. Furthermore, neutralization of IL-12 did not impact parasitemia (Fig 7G).

**Fig 8:**
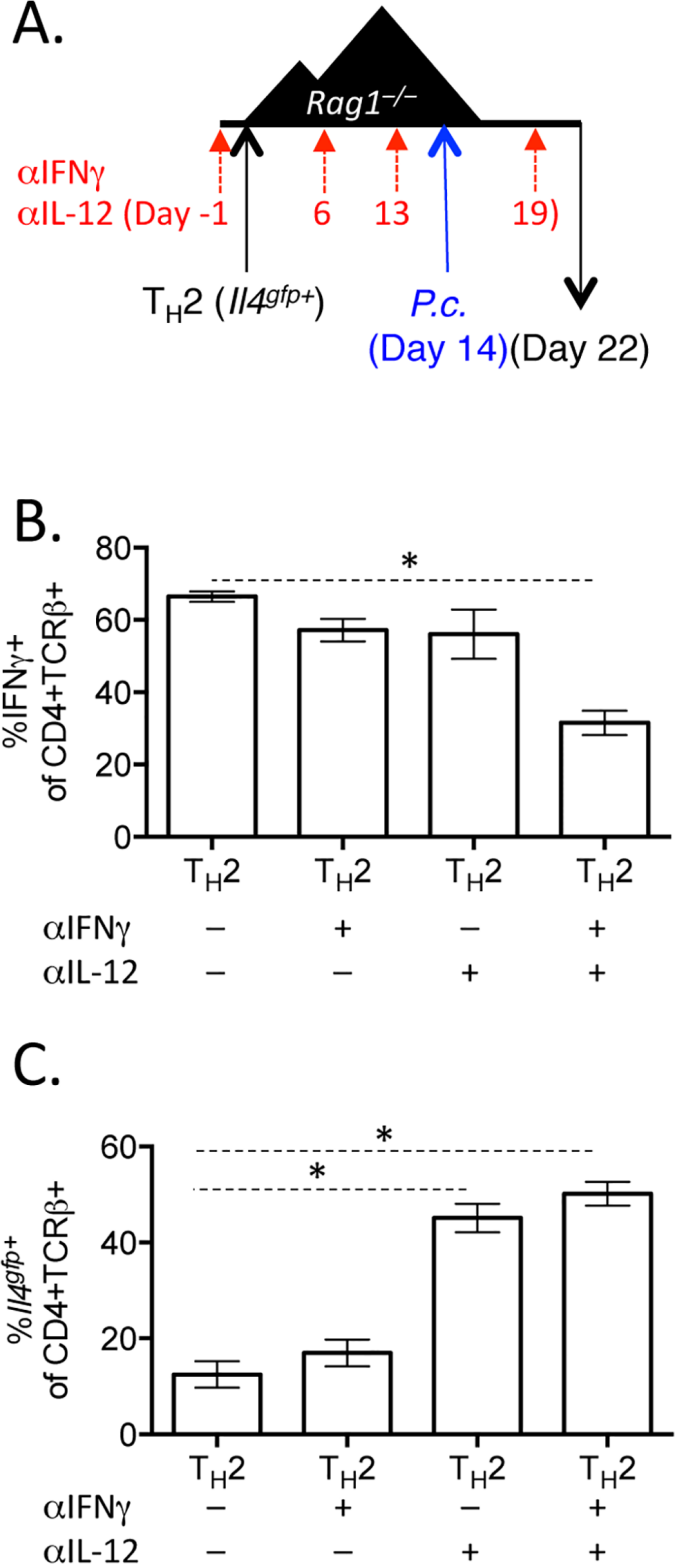
Blockade of IL-12 and IFNγ prevents optimal IFNγ production by Th2 cells. A–C). *In vitro* Th2 (CD4^+^TCRβ^+^ *Il4* ^gfp+^) cells were transferred to *Rag1* ^*–/–*^recipient mice for 14 days. Mice were infected with *P*. *chabaudi* and harvested at d8 post-infection. Mice were treated i.p. with 0.5mg anti-IL12 and anti-IFNγ at days -1, 6, 13, and 19. B). IFNγ production by transferred Th2 cells in the spleen, as determined by ICS. C). *Il4* ^*gfp*^ expression in transferred cells in the spleen. Data representative of 2 independent experiments with 5 mice per group. * denotes P<0.05.

We next tested whether IFNγ, which contributes to Th1 differentiation [48], was required for IFNγ expression by Th2 cells. To do this, we blocked IFNγ, IL-12, or both IFNγ and IL-12 throughout the experiment (Fig 8A). Blockade of IFNγ or IL-12 alone did not have a major impact on IFNγ production by Th2 cells (Fig 8B). As above, IL-12 blockade preserved *Il4* ^*gfp*^ expression in a population of Th2 cells (Fig 8C). However, blockade of both IFNγ and IL-12 led to a >50% reduction in IFNγ-expressing cells deriving from Th2 cells (from 66.7%±1.5% IFNγ^+^ cells to 31.6%±3.4% IFNγ^+^ cells, Fig 8B), indicating that both IL-12 and IFNγ were required for optimal conversion of Th2 cells into IFNγ-secreting cells during *Plasmodium* infection. Despite a 50% reduction in IFNγ-secreting cells following IL-12 and IFNγ blockade, the remaining ~30% of IFNγ^+^ cells were sufficient to prevent high parasitemia (S5 Fig).

### Blockade of IL-12 and IFNγ during helminth and *Plasmodium* co-infection preserves Th2 responses

**Fig 9:**
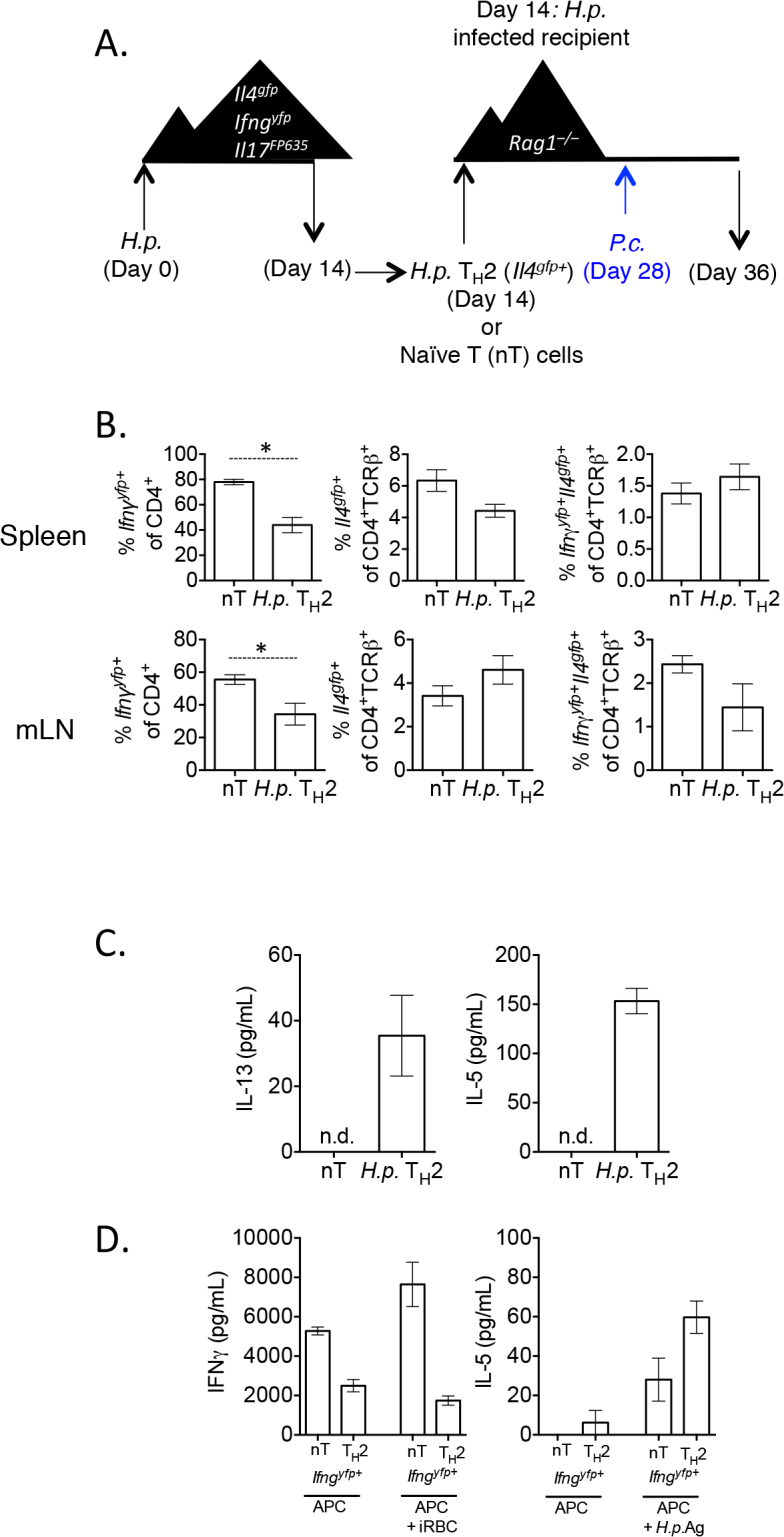
*H*. *polygyrus*-induced Th2 cells retain capacity to produce IFNγ during co-infection. A)–B). CD4^+^TCRβ^+^ *Il4* ^*gfp+*^ *Ifng* ^*yfp-*^ *Il17a* ^*FP635-*^ *ex vivo* Th2 cells from d14 *H*. *polygyrus-*infected mice or naïve CD4^+^ T cells were transferred to d14 *H*. *polygyrus*-infected *Rag1* ^*–/–*^recipient mice. Mice were infected with *P*. *chabaudi* and analyzed at day 8 post-infection. B). Cytokine reporter expression in transferred cells from spleens and mesenteric lymph nodes of recipient mice. Data representative of 2 separate experiments, with 4 mice per group. C). *Ex vivo* Th2 or naïve cells were transferred to recipient *Rag1* ^*-/-*^ mice, as in 9A. At day 8 post-infection with *P*. *chabaudi*, 2x10^5^ mesenteric lymph nodes cells were stimulated with 10 μg/mL *H*. *polygyrus* antigen and 10 ng/mL IL-4. ELISAs were performed on cell supernatants after 5 days. Data are representative of 2 separate experiments. Lymph nodes were pooled from 2 recipient mice per group. Error bars represent technical replicates. D). *Ex vivo* Th2 or naïve cells were transferred to recipient *Rag1* ^*-/-*^ mice, as in 9A. At day 8 post-infection with *P*. *chabaudi*, CD4^+^TCRβ^+^ *Ifng* ^*yfp+*^ *Il4* ^*gfp-*^ *Il17a* ^*FP635-*^ cells were sorted from pooled spleens of 2 recipient mice per group (following the sorting strategy as in Fig 2G). 9.6x10^4^ purified converted CD4^+^TCRβ^+^ *Ifng* ^*yfp+*^ *Il4* ^*gfp-*^ *Il17a* ^*FP635-*^ cells from a naïve or Th2 past were then cultured with 4x10^5^ irradiated CD4^+^–depleted splenocytes and one of the following: 3x10^6^ red blood cells from a *P*. *chabaudi*-infected donor mouse (day 8 post-infection) or 10 μg/mL *H*. *polygyrus* antigen. ELISAs were performed on cell supernatants after 5 days. Error bars represent technical replicates. Data are representative of 2 independent experiments.

Finally, we translated these new observations back into a co-infection scenario, as presented in Fig 1, and tested whether helminth-induced Th2 cells had the capacity to up-regulate IFNγ in a co-infection scenario. First, we purified *ex vivo Il4* ^*gfp+*^ *Ifng* ^*yfp–*^ *Il17a* ^*FP635–*^ Th2 cells from d14 *H*. *polygyrus*-infected mice and transferred them into day 14 *H*. *polygyrus*-infected *Rag1* ^*–/–*^mice. Recipient mice were then co-infected with *P*. *chabaudi* and the transferred cells were analyzed at day 8 post *P*. *chabaudi* infection (Fig 9A). Similar to *in vitro*-derived Th2 cells, *H*. *polygyrus-*derived Th2 cells down-regulated *Il4* ^*gfp*^ and up-regulated *Ifng* ^*yfp*^, albeit to a slightly lesser extent than naïve T cells (Fig 9B).

Re-stimulation of lymph node cells with *H*. *polygyrus* antigen and IL-4 led to the secretion of IL-5 and IL-13 from mice given *H*. *polygyrus* Th2 cells, but not from mice given naïve T cells (Fig 9C). These data suggested that despite a high degree of conversion to IFNγ-secreting cells, cells retained antigen-associated cytokine secretion. To more accurately determine whether converted cells retained the capacity to produce Th2 cytokines in an antigen-specific manner, we sorted Th2 cells, or naïve cells, that had converted into *Ifng* ^*yfp+*^ cells from recipient mice and restimulated them *in vitro* with *H*. *polygyrus* antigen or *P*. *chabaudi* infected red blood cells (iRBC). *Ifng* ^*yfp+*^ cells, which were previously naïve or *Il4* ^*gfp+*^ Th2 cells, produced IFNγ when co-cultured with irradiated APCs, supporting the cytokine reporter expression (Fig 9D). iRBCs further stimulated more IFNγ from naive T cells, but not from Th2 cells, suggesting that either *ex vivo* Th2 cells were not responding to malarial antigens, or that they were already secreting IFNγ at capacity. In addition, *ex vivo H*. *polygyrus* elicited Th2 cells which had down-regulated *Il4* ^*gfp*^ and up-regulated *Ifng* ^*yfp*^ produced IL-5 in response to *H*. *polygyrus* antigen, suggesting that converted cells retained antigen specificity and plasticity in this model (Fig 9D).

**Fig 10:**
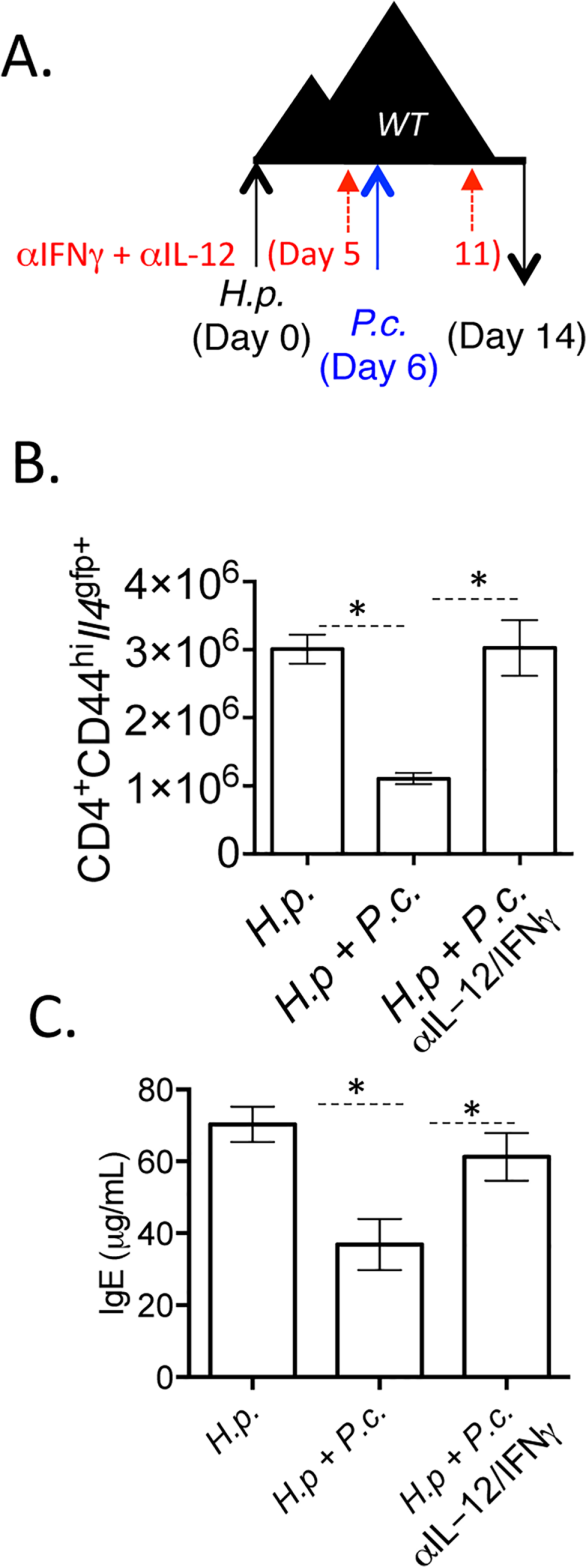
Blockade of IL-12 and IFNγ during co-infection preserves Th2 responses. A-C). C57BL/6 mice were orally infected with 200 *H*. *polygyrus* larvae. 6 days post-infection, mice were infected with 10^5^ *P*. *chabaudi*. At day 8-post infection with *P*. *chabaudi* (d14 *H*. *polygyrus*), mice were harvested. Mice were treated with 0.5 mg of anti-IL-12 and anti-IFNγ i.p. at days 0, 5, and 11. B). Total numbers of CD4^+^CD44^hi^ *Il4* ^gfp+^ cells in the mesenteric lymph nodes. C). IgE measured in the serum by ELISA. Data are representative of 2 independent experiments with 6 mice per group. * denotes P<0.05.

Finally, we tested whether the factors promoting IFNγ in the adoptive transfer model, IL-12 and IFNγ (Fig 8B), were responsible for the loss of Th2 cells and type-2 immunity during *H*. *polygyrus* and *P*. *chabaudi* co-infection. To do this, we infected wild type mice with *H*. *polygyrus* and at six days post-infection, mice were co-infected with *P*. *chabaudi* with or without blocking antibodies to IL-12 and IFNγ (Fig 10A). Blockade of IL-12 and IFNγ preserved *Il4* ^*gfp+*^ Th2 cells in co-infected mice (Fig 10B) and maintained elevated levels of helminth-induced type-2-associated IgE (Fig 10C). However, despite preserving Th2 cells and IgE, proficient anti-helminth immunity was not fully restored in mice given blocking antibodies (S6 Fig). Thus, IL-12 and IFNγ play a major role compromising Th2 responses during helminth/ *Plasmodium* co-infection, but additional factors also contribute to compromised anti-helminth immunity during co-infection.

## Discussion

In this study, we identified that *Plasmodium* infection significantly reduced CD4^+^ Th2 cells during co-infection with *H*. *polygyrus* and that anti-helminth immunity was compromised during co-infection. Mechanistically, we found that *Il4* ^*gfp+*^ *Ifng* ^*yfp–*^ *Il17a* ^*FP635–*^ Th2 cells, purified from novel triple cytokine reporter mice, converted to IFNγ-secreting cells, contributing significantly to anti-*Plasmodium* immunity. IFNγ production by Th2 cells was dependent on TCR, IL-12, and IFNγ signaling, all of which contributed to the transcriptional re-programming of Th2 cells. Finally, we found that blockade of IL-12 and IFNγ during *Plasmodium* and helminth co-infection preserved Th2 responses and IgE production, but was insufficient to fully restore anti-helminth immunity.

There is a large body of literature describing the prevalence of helminth and *Plasmodium* co-infection in human populations [4,5,8,11,49,50], and mouse models [16,51], with the majority of studies focusing on the impact of helminth infections on anti-*Plasmodium* responses. Relatively few have focused on how parasite-elicited Th2 responses are affected during *Plasmodium* co-infection. Our data show that IL-4-expressing Th2 cells, serum IgE, and functional parasite expulsion are reduced during co-infection (Fig 1). This is in line with previous reports, including reduced schistosome-specific IL-4 and IL-5 in *Plasmodium* and schistosome co-infected individuals [52] and suppressed IL-4 responses during *H*. *polygyrus* and *Plasmodium yeolii* co-infection [53]. Reduced type-2 responses [54] and Th2-mediated immunopathology have also been observed in schistosome and *Plasmodium* co-infected mice [55], consistent with the notion that anti-helminth associated Th2 responses are compromised during *Plasmodium* co-infection. However, these studies did not offer mechanistic insight as to how this reduction in type-2 immunity might occur and importantly how type-2 immunity might be preserved during co-infection.

In this study, we focused on the impact of co-infection on CD4^+^ T cells, which are a critical cell type for immunity to *H*. *polygyrus* and contribute significantly to anti-malarial immunity [56]. For our studies, we developed a triple cytokine reporter mouse (*Il4* ^*gfp*^ *Ifng* ^*yfp*^ *Il17a* ^*FP635*^, S1 Fig), which had several important advantages. These mice allowed the determination of T cell phenotype *ex vivo* without the need for re-stimulation, as well as the ability to obtain highly purified populations of *Il4* ^*gfp+*^ *Ifng* ^*yfp–*^ *Il17a* ^*FP635–*^ Th2 cells, which were not expressing other lineage-associated cytokines[29]. Adoptive transfer of these cells allowed us to accurately determine whether purified Th2 cells changed their phenotype, and finally, simultaneous cytokine reporters allowed us to test whether any conversion was reversible and truly plastic. To this end, we observed that highly-purified *Il4* ^*gfp+*^ *Ifng* ^*yfp–*^ *Il17a* ^*FP635–*^ Th2 cells, either generated *in vitro* for two weeks (Fig 2) or isolated *ex vivo* from *H*. *polygyrus*-infected mice (Fig 9), were able to produce IFNγ during *Plasmodium* infection in *Rag1* ^*–/–*^mice. This phenomenon is in line with several previous observations 1) identifying that *in vitro* generated LCMV-specific TCR transgenic Th2 cells could express both IFNγ and IL-4 [34], 2) a ‘bi-functional’ population of Tbet^+^ GATA3^+^ cells are generated following *H*. *polygyrus* infection [29] and 3) the *Tbx21* locus (encoding T-bet) has bivalent epigenetic histone modifications in Th2 cells [57] suggesting Th2 cells retain some flexibility. We observed expression of *Ifng*, *Tbx21*, *Klrg1*, *Gzmb*, *Gzmc* in converted Th2 cells, while maintaining low levels of *Il4* transcription (Fig 3, S1 Table) and the ability to produce IL-5 and IL-13 (Fig 2). This suggested that converted cells were possibly poly-functional. Whether they are similar to ‘bi-functional’ cells [29] is unclear. Helmby observed exacerbated liver pathology with significantly increased IFNγ and mortality during *H*. *polygyrus* and *Plasmodium* co-infection [58]. Whether Th2 cells converted to IFNγ-secreting cells, contributing to aggravated liver pathology in their study was unclear. Similarly, Th2 cells that up-regulate IL-17 during airway allergen challenge in mice contribute to more severe airway pathology [59], and allergic patients have a greater frequency of IFNγ-secreting cells [60]. Indeed, polyfunctional T cells, which secrete multiple cytokines, correlate with greater protection following vaccination [61], contribute to severe inflammatory syndromes in humans [62] and mice [37] and have greater anti-tumor activity [63]. Thus, understanding the mechanisms of Th cell conversion and the generation of polyfunctional T cells may provide important insight into immunity and immunopathology. Interestingly, in our model of *C*. *albicans*, *in vitro* polarized Th2 cells were unable to produce IL-17a, unlike naïve cells (S4 Fig), suggesting that there is either an important relationship between Th2 and Th1 cells, or that the transcriptional machinery required for IL-17 production is more tightly regulated than for IFNγ.

To identify mechanistic pathways contributing to Th2 cell conversion, we employed RNA-Seq analysis of Th1 cells (*Ifng* ^*yfp+*^), Th2 cells (*Il4* ^*gfp+*^) and Th2 cells that had up-regulated IFNγ (*Ifng* ^*yfp+*^ *Il4* ^*gfp–*^). We identified a high degree of transcriptional similarity between Th1 cells and converted cells, extending significantly beyond cytokine expression. For example, Th1 and converted *Ifng* ^*yfp+*^ *Il4* ^*gfp–*^cells, but not Th2 cells, had similar transcript abundance encoding for several enzymes (*Bace2*, *Cdc25c*, *Cd38 Chst11*, *Dusp5*, *Gzmb*, *Gzmc and Gzmk*, *Gstt1*, *Pdcd1*, *Ptpn5*, *Spag5*, *Troap*), chemokine receptors (*Cxcr3*, *Cmklr1*, *Cx3cr1 and Ccr5*), ion channels (*Cacna1l and Ttyh2*), kinases (*Stk32c*, *Ttbk1*, *Ttk*, *Ltk*, *Cdk1*, *Pbk*, *Ccnb1* in addition to many other kinases), nuclear receptors (*Nr4a2*, *Ahr*), miRNAs (*miR-142*, *miR-155 and miR-Let7d*) and transcriptional regulators (*Rai14*, *E2f7*, *Gas7*, *Cdkn2b*, *E2f8*, *Klf12*, *Runx2* and *Eomes*). Significantly, Th1 cells use a feed-forward regulatory circuit involving *Tbx21* (Tbet) and *Runx3* for maximal IFNγ production and silencing of *Il4* [64]. In our study, both Th1 cells and converted Th2 cells which had lost *Il4* and up-regulated *Ifng*, had elevated *Runx3* and *Tbet*, suggesting that this feed-forward loop was transcriptionally active, supporting optimal IFNγ production in converted cells. Whether the epigenetic landscape of converted cells matched that of their Th1 counterparts is of great interest, as converted Th2 cells retained the capacity to produce Th2-associated IL-5 and IL-13 (Fig 2) in an antigen-specific manner (Fig 9D). Previous studies have indicated that Th1 cells have the capacity to up-regulate Th2-associated features *in vivo* following helminth infection [65]. In our hands, naïve T cells which had up-regulated IFNγ^+^ *in vivo* following *Plasmodium* infection did not have the capacity to secrete IL-5 or IL-13 when re-stimulated *in vitro* with anti-CD3/28 and IL-4. Whether there are specific *in vivo* factors which more readily support T cell plasticity is currently unclear. We would hypothesize that *in vitro* generated or *ex vivo H*. *polygyrus* Th2 cells had bivalent methylation marks in the *Il5* and *Il13* locus allowing re-expression of these genes following the appropriate activating signal. Supporting this, converted Th2 cells retained some Th2-associated features, including elevated expression of *Gfi1*, *Il4* and *Il33r*, which may provide the appropriate machinery to re-activate Th2-associated genes, reminiscent of their Th2 past (Fig 3 and S1 Table).

Using an upstream analysis algorithm (Ingenuity Pathways Analysis) with our transcriptional data sets we identified IL-12, IFNγ and to a lesser extent type 1 IFN, as putative factors that could contribute to the observed transcriptional profile of converted cells. This supports a recent study that identified the requirement of Tbet and Stat4 for IFNγ expression in memory Th2 cells [66]. In our study, unlike previous studies, type I IFN signalling in Th2 cells was dispensable for IFNγ production from converted Th2 cells *in vivo* (Fig 6) [34]. Blocking IL-12 or IFNγ alone did not impact the frequency of converted IFNγ^+^ cells from transferred Th2 cells (Figs 7 and 8). These data are in agreement with a previous study that found restoring IL-12 responsiveness in Th2 cells, through ectopic expression of IL-12Rβ2, was insufficient to convert Th2 cells into IFNγ-secreting cells [67]. However, in our model, anti-IL-12 treatment alone preserved IL-4 expression in a sub-population of transferred cells (Figs 7 and 8). Blockade of both IFNγ and IL-12 substantially reduced IFNγ^+^ cells deriving from Th2 cells, suggesting that an IL-12-STAT4 signaling pathway down-regulated IL-4, while an IFNγ / STAT-1 / T-bet pathway was required for optimal IFNγ expression, in accordance with canonical Th1-inducing conditions for naive T cells [68]. While we found that blockade of these cytokines reduced IFNγ^+^ cells, there was no change in control of parasitemia (S5 Fig). We speculate that this is due to the incomplete loss of conversion, with the remaining IFNγ being sufficient to control levels of parasitemia.

TCR stimulation was essential for *in vitro*-derived Th2 cells to produce IFNγ (Fig 5) and *ex vivo H*. *polygyrus-*elicited Th2 cells required *H*. *polygyrus*-infected recipient mice to survive and up-regulate IFNγ. Thus, with sufficient TCR signaling, a change in the local cytokine milieu may be sufficient to re-program Th cells. During helminth and *Plasmodium* co-infection, either cross-reactive antigens or microflora-derived signals may provide the necessary first TCR signal [69–71]. Alternatively the broad polyclonal activation of non-specific T cells during *Plasmodium* infection may be sufficient [21,22,72]. Although TCR engagement, IL-12 and IFNγ were required for optimal conversion of Th2 cells into IFNγ-secreting cells, it is possible that other factors also contribute to conversion, including IL-27, which can induce expression of Tbet, and IL-18, which can induce IFNγ production [73,74].

In conclusion, we have shown that IL-12 and IFNγ suppressed Th2 responses during *H*. *polygyrus and P*. *chabaudi* co-infection. Mechanistically, we identified that TCR engagement with IL-12 and IFNγ signaling converted *in vitro*-generated Th2 cells into IFNγ-producing cells during *P*. *chabaudi* infection. Importantly, although blocking IL-12 and IFNγ during co-infection did not retain fulminant anti-helminth immunity, it did preserve Th2 cell numbers and serum IgE, highlighting a novel mechanistic pathway of how *Plasmodium* infection negatively impacts anti-helminth Th2 responses. Overall, our studies indicate that *Plasmodium* infection can negatively impact anti-helminth responses, that Th2 cells retain substantial plasticity in the context of *Plasmodium* infection, and that this plasticity may play a role in the reduced Th2 response during co-infection.

## Materials and Methods

### Animals

All mice were bred and maintained under specific pathogen-free conditions at the National Institute for Medical Research. Strains used included: C57BL/6, *Ifng* ^*yfp*^ [36], *Il4* ^*gfp*^[35], C57BL/6 *Rag1* ^*–/–*^[75], *MhcII* ^*–/–*^(B6.129-H-2<dlAb1-Ea)[76] crossed with *Rag1* ^*–/–*^at NIMR [77], OTII *Rag1* ^*–/–*^(B6.Cg (Tcrαβ)425Cbn/J) [78], OTII *Il4* ^*gfp*^ *Rag1* ^*–/–*^(OTII *Rag1* ^*–/–*^crossed with *Il4* ^*gfp*^ at NIMR), and *Ifnar* ^*–/–*^ *Il4* ^*gfp*^ (*Ifnar* ^*–/–*^[79] crossed with *Il4* ^*gfp*^ at NIMR). Triple cytokine reporter mice (*Il4* ^*gfp*^ *Il17* ^*Cre*^ *Ifng* ^*yfp*^R26^FP635^) were established by crossing *Il4* ^*gfp/gfp*^ *Il17* ^*Cre/Cre*^[37] mice with *Ifng* ^*yfp/+*^ *R26* ^*FP635*/FP635^ mice, producing *Il4* ^*gfp/+*^ *Il17* ^*Cre/+*^ *Ifng* ^*yfp/+*^R26^FP635/+^. The generation of *R26* ^*FP635*^ reporter mice will be presented in detail elsewhere (JB and AP, manuscript in preparation). Briefly, *R26* ^*FP635*^ mice were generated by inserting the coding sequences of the red fluorescent protein FP635 [80] into the pROSA26 targeting vector downstream of a loxP-flanked neomycin resistance cassette containing three transcriptional stop signals by homologous recombination. *R26* ^*FP635*^ reporter mice in this study were backcrossed to C57BL/6 for more than 8 generations.

### Infections

Mice were infected by oral gavage with 200 infective stage 3 (L3) *Heligmosomoides polygyrus* larvae, diluted in water. The anthelmintic drug pyrantel pamoate (Sigma, 5mg/dose in water) was given orally on two consecutive days. Infections with *Plasmodium chabaudi chabaudi* (AS) were performed by i.p. injection of 10^5^ parasitized red blood cells. Parasitemia was measured by blinded counting of Giemsa-stained blood smears. Anemia and hemoglobin were measured by diluting blood in Krebs buffered saline with 0.2% glucose and with 100 IU/mL heparin and measured using Vetscan (Abaxis-VetScan HM5 Hematology). Infections with *Candida albicans* were performed by i.v. injection of 10^5^ yeast forms.

### Cell sorting and flow cytometry

Cell sorting was performed using a FACS Aria II (BD Biosciences), MoFlo XDP (Beckman Coulter), or Influx (BD Biosciences) cell sorter. To prepare cells for sorting, CD4^+^ cells were first positively selected using MACS CD4 beads and magnetic columns (Miltenyi Biotec). Cell suspensions were then stained for 25 minutes with antibodies in PBS with 1% FCS. To prepare for sorting, stained cells were diluted in phenol-red free IMDM (Gibco) (with 1% FCS, 2mM EDTA (Invitrogen), 100 U/mL Penicillin and 100 μg/mL Streptomycin (Gibco), 8 mM L-glutamine (Gibco), and 0.05 mM 2-mercaptoethanol (Gibco)). Propidium iodide (PI) was used to determine cell viability in sorting experiments. Intracellular cytokine staining (ICS) was performed following 6 hours of re-stimulation with 50ng/mL phorbol 12-myristate 13-acetate (PMA, Promega) and 1 μg/mL ionomycin (Sigma) and BD Golgi Stop and BD Golgi Plug (diluted 1:1000, BD Biosciences). Following surface stain, cells were incubated with eBioscience Fixation/Permeabilization buffer for 25 minutes followed by 25 minutes in Permeabilization buffer (eBioscience), and incubation with antibodies in Permeabilization buffer for a further 30 minutes. For flow cytometry analysis, cells were analyzed using a BD LSRII (BD Biosciences) and data were analyzed using FlowJo software (Version 7.6.5, Treestar Inc). In all cases using triple cytokine reporter mice, cells from wild type, *Ifng* ^*yfp*^ or *Il4* ^*gfp*^ single cytokine reporter mice were used as controls to set gates to differentiate yfp and gfp. Antibodies used include: CD4 (efluor450 and PE-Cy7, RM4-5, eBioscience), CD25 (Fitc, 7D4, BD Pharmingen), CD44 (Fitc, Percpcy5.5, and APC, IM7, eBioscience), CD45.1 (PE-Cy7 and APC, A20, eBioscience), IFNγ (Pacific Blue, XMG1.2, Biolegend), IL4 (PE, 11B11, eBioscience), pSTAT4 (Alexa Fluor 647, BDPhosflow), TCRβ (APC, H57-597, eBioscience) and GFP (Alexafluor647, FM264G, BioLegend). Staining was performed in presence of FcR Blocking Reagent (Miltenyi Biotec). In analysis experiments, viability was determined using the Molecular Probes Live/Dead Fixable Blue Dead Cell Stain Kit (Life Technologies). For phospho-STAT staining, sorted cells were resuspended into serum-free media and incubated at 37 degrees for 20 minutes, followed by incubation with 10 ng/mL IL-12 (R&D) for 15 minutes. Cells were then fixed for 10 minutes at 37 degrees with prewarmed BD Phosflow Lyse/Fix Buffer, washed, permeabilized with BD Phosflow Perm Buffer III for 30 minutes on ice, washed, and stained for 1 hour with antibodies in PBS for FACS analysis.

### Adoptive cell transfer

Naive CD4^+^ T cells were sorted from spleens as CD4^+^TCRβ^+^CD44^–^CD25^–^ *Il4* ^*gfp*–^PI^−^(*Il4* ^*gfp*^ reporter) or CD4^+^TCRβ^+^CD44^–^CD25^–^ *Il4* ^*gfp-*^ *Ifng* ^*yfp–*^ *Il17a* ^*FP635*–^PI^−^(triple reporter). Th2 cells were cultured for 2 weeks from splenic CD4^+^ cells *in vitro* with 10 ng/mL IL-4 (R&D), 5 ng/mL IL-2 (R&D), 10 μg/mL anti-IFNγ (XMG1.2, BioXcell), and Mouse T-Activator CD3/CD28 Dynabeads (Life Technologies) in IMDM with 10% FCS. Th2 cells were sorted as CD4^+^TCRβ^+^ *Il4* ^*gfp*+^PI^−^(*Il4* ^*gfp*^ reporter) or CD4^+^TCRβ^+^ *Il4* ^*gfp+*^ *Ifng* ^*yfp–*^ *Il17a* ^*FP635*–^PI^−^(triple reporter). For each experiment, 0.2x10^6^ to 1x10^6^ cells were adoptively transferred i.v. into recipient C57BL/6 *Rag1* ^*–/-*^ mice. Blocking antibodies diluted in PBS (anti-IFNγ, XMG1.2, anti-IL12p40 C17.8, BioXcell) were used at 0.4 or 0.5 mg/ dose.

### Cell restimulation and ELISA

Sorted cells were cultured in 96 well round bottom plates in various conditions. Where indicated, antigen presenting cells were spleens depleted of CD4^+^ cells by MACS magnetic separation (Miltenyi Biotec) and irradiated (3000 rads). *H*. *polygyrus* antigen was isolated by homogenization of cleaned adult worms in PBS. IFNγ, IL-5, and IL-13 were measured using DuoSet ELISA kits, according to the manufacturer’s instructions (R&D). Total IgE ELISA was performed by coating with Purified Rat Anti-Mouse IgE (R35-72, BD Pharmingen) at 2 μg/mL overnight, followed by overnight incubation with serum and standard (Purified Mouse IgE,k isotype Standard, BD Pharmingen), and detection with Biotin Rat Anti-Mouse IgE at 1 μg/mL (R35-118, BD Pharmingen), Streptavidin HRP at 1:000 (BD Pharmingen) and ABTS One Component HRP Microwell Substrate (SurModics). *H*. *polygyrus-*specific IgG1 was detected by coating plates with 5 μg/mL *H*. *polygyrus* antigen overnight, followed by overnight incubation with serially diluted serum and detection with Biotin Rat Anti-Mouse IgG1 (Invitrogen) and streptavidin and ABTS, as above.

### RNA extraction, qRT-PCR, RNA-Seq and IPA analysis

RNA was isolated from cells or tissue using RNeasy Mini Kit according to manufacturer’s instructions (Qiagen). For qRT-PCR of small intestine-derived RNA, 1 cm sections of tissue were harvested and stored in RNAlater (Sigma) before homogenisation and RNA extraction using RNeasy Mini Kit (Qiagen). cDNA was reverse transcribed from RNA using QuantiTect Reverse Transcription Kit (Qiagen) according to the manufacturer’s instructions. qRT-PCR analysis was performed using Power SYBR Green PCR master mix (Applied Biosystems) on an ABI Prism 7900HT Sequence Detection System (Applied Biosystems). Relative quantities of mRNA were determined by the comparative threshold cycle method as described by Applied Biosystems for the ABI Prism 7700/7900HT Sequence Detection Systems using the following primers; *Hprt* Fwd: 5’-GCCCTTGACTATAATGAGTACTTCAGG-3’ and Rvs: 5’-TTCAACTTGCGCTCATCTTAGG-3’; *Retnla* Fwd: 5’-CCCTCCACTGTAACGAAGACTC-3’

and Rvs: 5’-CACACCCAGTAGCAGTCATCC-3’; *Chil3*: Fwd: 5’- CATGAGCAAGACTTGCGTGAC-3’ and Rvs: 5’-GGTCCAAACTTCCATCCTCCA-3’; *Arg1* Fwd: 5’- GGAAAGCCAATGAAGAGCTG -3’ and Rvs: 5’- GCTTCCAACTGCCAGACTGT -3’. RNA-seq libraries were constructed using the TruSeq RNA Sample Preparation Kit V2 according to manufacturer’s instructions (Illumina). Libraries were sequenced using the HiSeq 2500 System (Illumina).The raw Illumina reads were analyzed as follows. First, the data quality was analyzed using FastQC (www.bioinformatics.babraham.ac.uk/projects/fastqc). Low quality bases were trimmed using Trimmomatic [81], and the read pairs which passed the trimming quality filters were aligned to mm10 (Ensembl version 75) using Tophat2 [82]. Counts were determined using htseq_count [83]. Normalisation and statistical analysis was performed using edgeR [84]. Statistically significant genes with FDR < 0.05 are reported. Significantly differentially expressed genes were uploaded into Ingenuity Pathways Analysis (IPA) and subjected to upstream analysis to identify factors that could have contributed to the transcriptional profile observed in converted Th2 cells.

### Statistical analysis

Data sets were compared by Mann Whitney test using GraphPad Prism (V.5.0). Differences were considered significant at *P ≤ 0.05.

### Ethics statement

All animal experiments were carried out following United Kingdom Home Office regulations (project license 80/2506) and were approved by UK National Institute for Medical Research Ethical Review Panel.

## Supporting Information

S1 Fig Generation of triple cytokine reporter mouse (*Il4* ^*gfp*^ *Ifng* ^*yfp*^ *Il17a* ^*FP635*^). A). Triple cytokine reporter mice were established by crossing *Il4* ^*gfp/gfp*^ *Il17a* ^*Cre/Cre*^ mice with *Ifng* ^*yfp/+*^ *R26* ^*FP635*/FP635^ mice, producing *Il4* ^*gfp/+*^ *Il17a* ^*Cre/+*^ *Ifng* ^*yfp/+*^R26^FP635/+^ genotypes, where ^+^ denotes wild type. B). CD4^+^ T cells from triple cytokine reporter mice were differentiated *in vitro* under Th2 conditions, as described in materials and methods. *Il4* ^*gfp+*^ *Il17a* ^*FP635–*^ *Ifng* ^*yfp–*^cells were FACS-purified for adoptive transfer, as described.
(TIF)

S2 Fig A). Wild type mice were co-infected with *H*. *polygyrus* and *P*. *chabaudi* as in Fig 1A. RNA was extracted from the small intestine and analyzed for expression of the macrophage alternative activation markers *Retnla* (Relmα/Fizz1), *Arg1*, and *Chil3* (Ym1) by real time PCR. Data represent 2 independent experiments with 2–5 mice per group. B and C). Triple reporter mice were co-infected with *H*. *polygyrus* and *P*. *chabaudi* as in Fig 1A. Total numbers of CD4^+^CD44^hi^ *Ifng* ^*yfp+*^ and *Il17a* ^FP635+^ cells in the mesenteric lymph nodes and spleen are shown. Data are representative of at least 2 experiments with 2–4 mice per group. D). Experimental set-up: *Il4* ^*gfp*^ reporter mice were orally infected with 200 *H*. *polygyrus* larvae followed by 10^5^ *P*. *chabaudi*-infected red blood cells at day 6 post-infection. Mice were harvested at day 28 post-infection. E). Total numbers of CD4^+^CD44^hi^ *Il4* ^*gf*p+^ cells in the mesenteric lymph nodes. F). IgE measured in the serum by ELISA. Data is representative of 2 independent experiments with 5 mice per group. G). Wild type mice were taken through the secondary co-infection model, as shown in Fig 1D. At day 15 post-infection, *H*. *polygyrus*-specific IgG1 in the serum was assessed by ELISA. Representative of 3 separate experiments, with 6 mice per group.
(TIF)

S3 Fig *In vitro* Th2 cells express *Ifng* ^*yfp*^ in the absence of *P*. *chabaudi* infection. A). Experimental set-up: 2-week *in vitro* polarized Th2 cells were FACS sorted as CD4^+^ *Il4* ^*gfp+*^ *Ifng* ^*yfp–*^ *Il17a* ^*FP635–*^ and transferred i.v. to *Rag1* ^*–/–*^mice. Recipient mice were infected with 10^5^ *P*. *chabaudi* i.p. on day 14 post-transfer or left uninfected. Mice were harvested at day 8 post-infection. B). Percent of CD4^+^TCRβ^+^ *Il4* ^*gfp+*^ and *Ifng* ^*yfp+*^ cells in the spleen, as determined by FACS. C). Total numbers of CD4^+^TCRβ^+^ *Il4* ^*gfp+*^ and *Ifng* ^*yfp+*^ cells in the spleen, as determined by FACS. Data are representative of 2 separate experiments, with 4–6 mice per group.
(TIF)

S4 Fig *In vitro* Th2 cells produce IFNγ but not IL-17a following infection with *C*. *albicans*. A). Experimental set-up: 2 week *in vitro* polarized Th2 cells were FACS sorted as CD4^+^ *Il4* ^*gfp+*^ and transferred i.v. to *Rag1* ^*–/–*^mice. As a control, a group of *Rag1* ^*–/–*^mice received naïve CD4^+^ T cells. Recipient mice were infected with 10^5^ *C*. *albicans* yeast forms i.v. on day 14 post-transfer and harvested at day 6 post-infection. B). Percent of CD4^+^TCRβ^+^ cells producing IFNγ, IL-17a, or GFP (IL-4) in the spleen, as determined by intracellular cytokine staining. Data are representative of 4 separate experiments, with 3–5 mice per group.
(TIF)

S5 Fig Blockade of IL-12 and IFNγ does not alter control of parasitemia in Th2 cell recipient mice. *In vitro* Th2 (CD4^+^TCRβ^+^ *Il4* ^*gfp*+^) cells were transferred to *Rag1* ^*–/–*^recipient mice for 14 days. Mice were infected with *P*. *chabaudi* and harvested at d8 post-infection. Mice were treated i.p. with 0.5mg anti-IL12 and anti-IFNγ at days -1, 6, 13, and 19, as shown in Fig 8A. Percent parasitemia was determined by blinded counting of Giemsa-stained blood smears. Data representative of 2 independent experiments with 3–5 mice per group. * denotes P<0.05.
(TIF)

S6 Fig Blockade of IL-12 and IFNγ during co-infection does not fully restore anti-helminth immunity. A). C57BL/6 mice were infected with 200 *H*. *polygyrus* larvae, treated on 2 consecutive days (days 16 and 17) with pyrantel embonate (5 mg), infected with 10^5^ *P*. *chabaudi* (day 31) and re-infected with *H*. *polygyrus* (day 38). Mice were treated with 0.5 mg of anti-IL-12 and anti-IFNγ i.p. at days 30, 36 and 40. B). Adult worms in intestine were counted on day 53. Data are representative of 2 independent experiments with 5–7 mice per group. * denotes P<0.05.
(TIF)

S1 Table Differentially expressed genes in Th1 (*Ifng* ^*yfp*+^), Th2 (*Il4* ^*gfp*+^) and Th2->*Ifng* ^*yfp+*^ cells. Normalized reads from RNA-Seq data were converted into fold-change values for analysis. Data are expressed relative to naive T cells, with the mean fold change derived from 3 biological replicates.
(PDF)
