# Supplementary figures and images for: IFNγ and IL-12 Restrict Th2 Responses during Helminth/Plasmodium Co-Infection and Promote IFNγ from Th2 Cells
Source: PLoS Pathog. 2015 Jul 6;11(7):e1004994. doi: 10.1371/journal.ppat.1004994 (PMC4493106; doi:10.1371/journal.ppat.1004994)

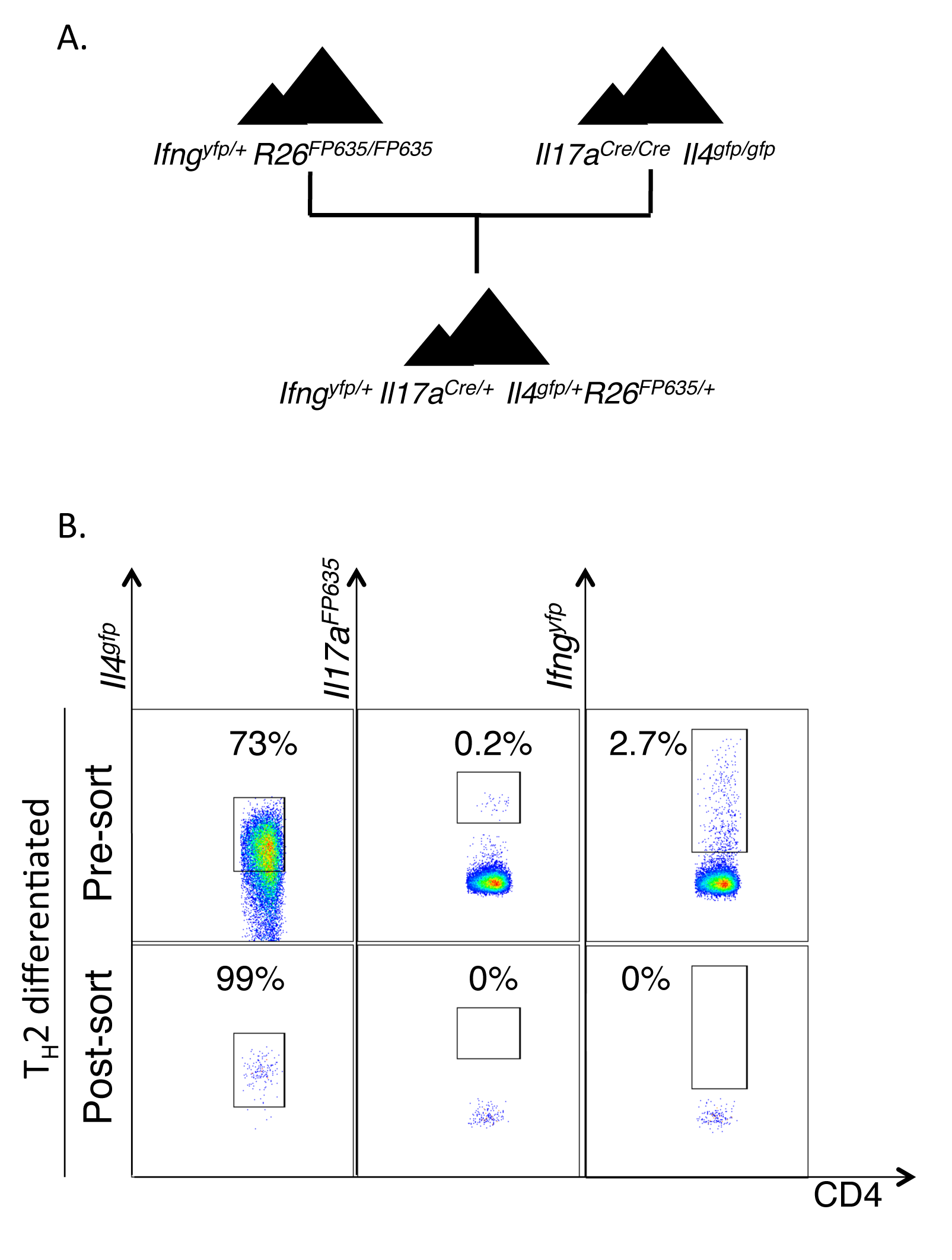

Supplement: S1 Fig — A). Triple cytokine reporter mice were established by crossing Il4 gfp/gfp Il17a Cre/Cre mice with Ifng yfp/+ R26 FP635/FP635 mice, producing Il4 gfp/+ Il17a Cre/+ Ifng yfp/+R26FP635/+ genotypes, where + denotes wild type. B). CD4+ T cells from triple cytokine reporter mice were differentiated in vitro under Th2 conditions, as described in materials and methods. Il4 gfp+ Il17a FP635– Ifng yfp–cells were FACS-purified for adoptive transfer, as described. (TIF) [file ppat.1004994.s001.tif]

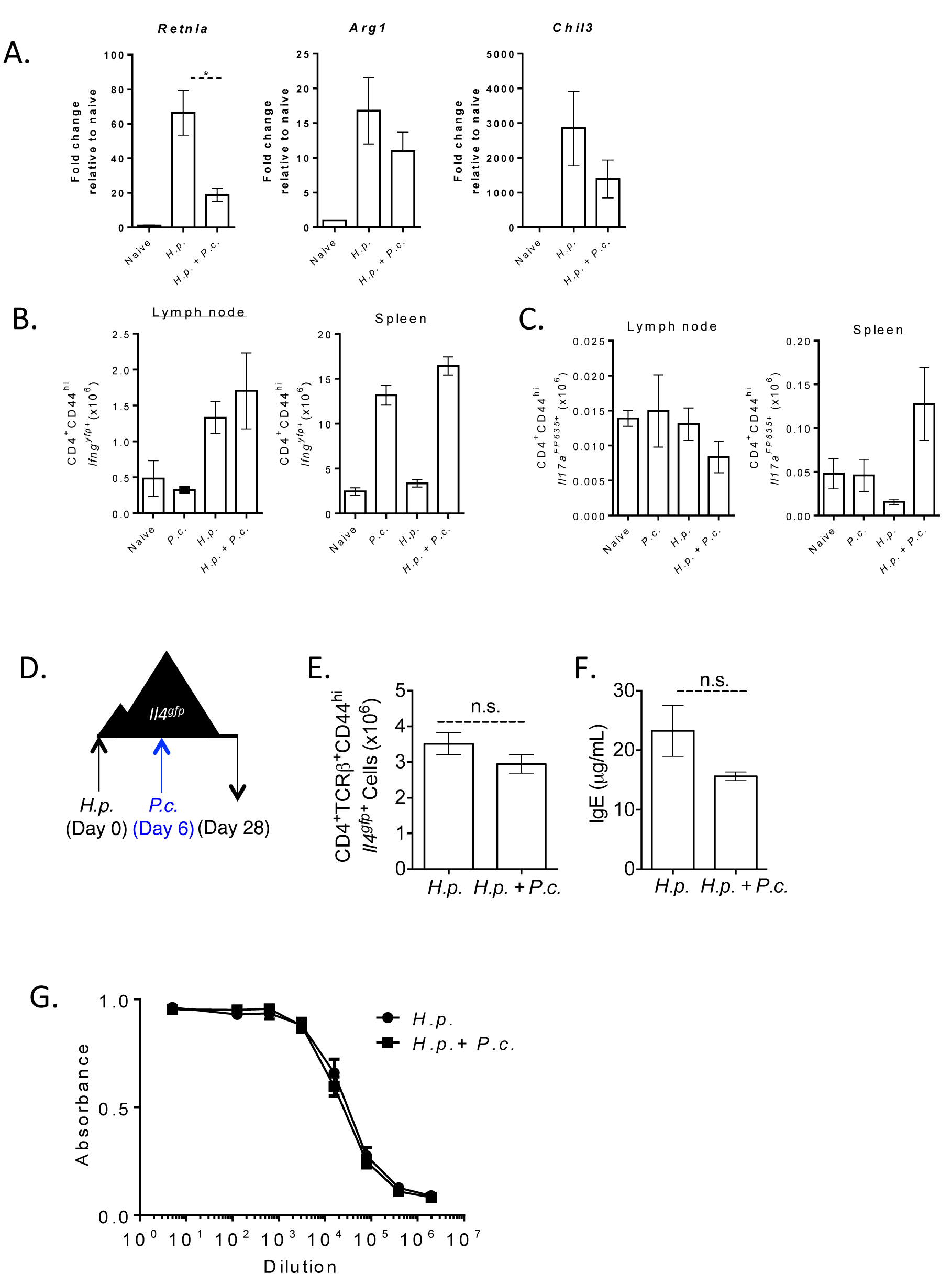

Supplement: S2 Fig — RNA was extracted from the small intestine and analyzed for expression of the macrophage alternative activation markers Retnla (Relmα/Fizz1), Arg1, and Chil3 (Ym1) by real time PCR. Data represent 2 independent experiments with 2–5 mice per group. B and C). Triple reporter mice were co-infected with H. polygyrus and P. chabaudi as in Fig 1A. Total numbers of CD4+CD44hi Ifng yfp+ and Il17a FP635+ cells in the mesenteric lymph nodes and spleen are shown. Data are representative of at least 2 experiments with 2–4 mice per group. D). Experimental set-up: Il4 gfp reporter mice were orally infected with 200 H. polygyrus larvae followed by 105 P. chabaudi-infected red blood cells at day 6 post-infection. Mice were harvested at day 28 post-infection. E). Total numbers of CD4+CD44hi Il4 gfp+ cells in the mesenteric lymph nodes. F). IgE measured in the serum by ELISA. Data is representative of 2 independent experiments with 5 mice per group. G). Wild type mice were taken through the secondary co-infection model, as shown in Fig 1D. At day 15 post-infection, H. polygyrus-specific IgG1 in the serum was assessed by ELISA. Representative of 3 separate experiments, with 6 mice per group. (TIF) [file ppat.1004994.s002.tif]

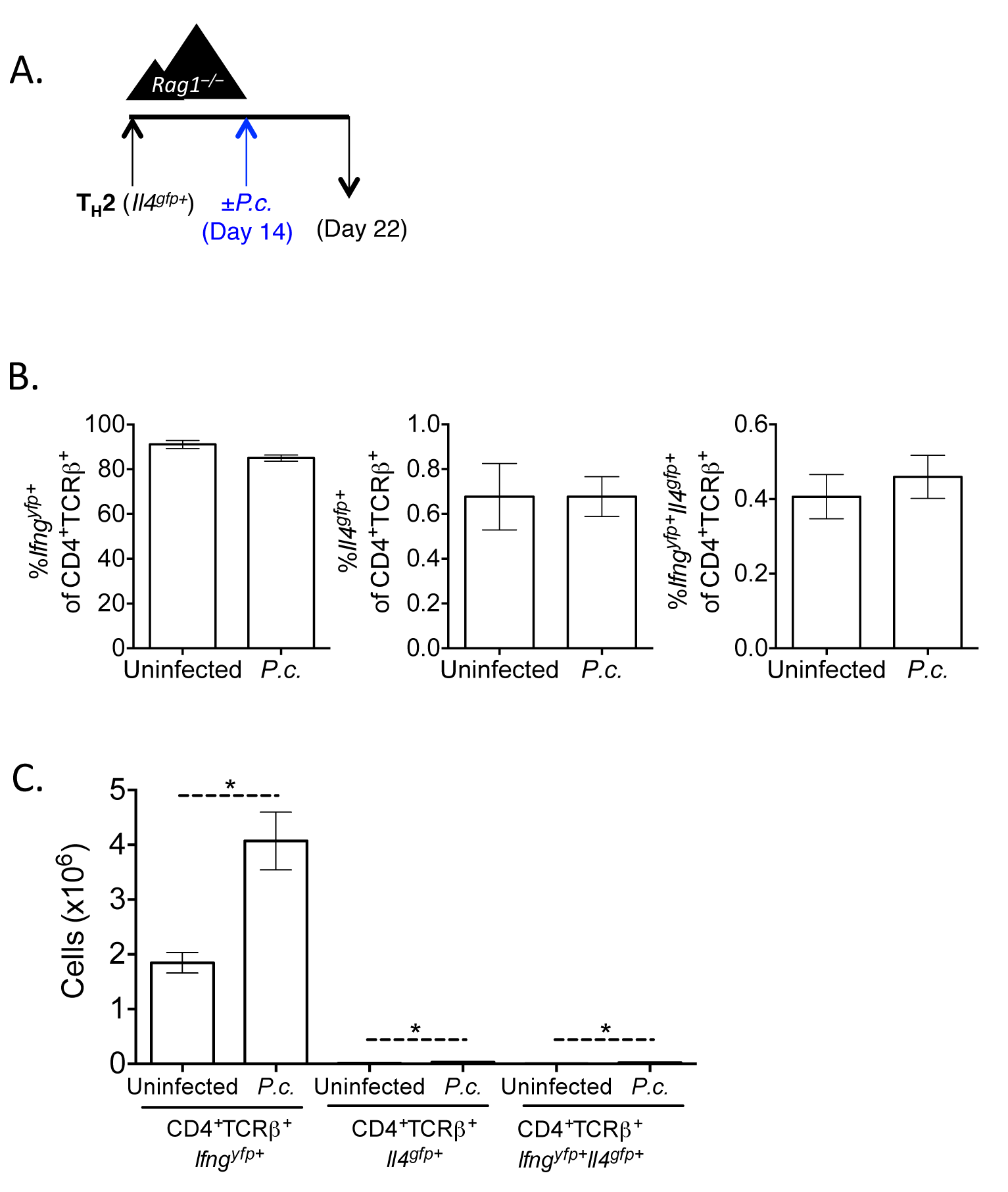

Supplement: S3 Fig — A). Experimental set-up: 2-week in vitro polarized Th2 cells were FACS sorted as CD4+ Il4 gfp+ Ifng yfp– Il17a FP635– and transferred i.v. to Rag1 –/–mice. Recipient mice were infected with 105 P. chabaudi i.p. on day 14 post-transfer or left uninfected. Mice were harvested at day 8 post-infection. B). Percent of CD4+TCRβ+ Il4 gfp+ and Ifng yfp+ cells in the spleen, as determined by FACS. C). Total numbers of CD4+TCRβ+ Il4 gfp+ and Ifng yfp+ cells in the spleen, as determined by FACS. Data are representative of 2 separate experiments, with 4–6 mice per group. (TIF) [file ppat.1004994.s003.tif]

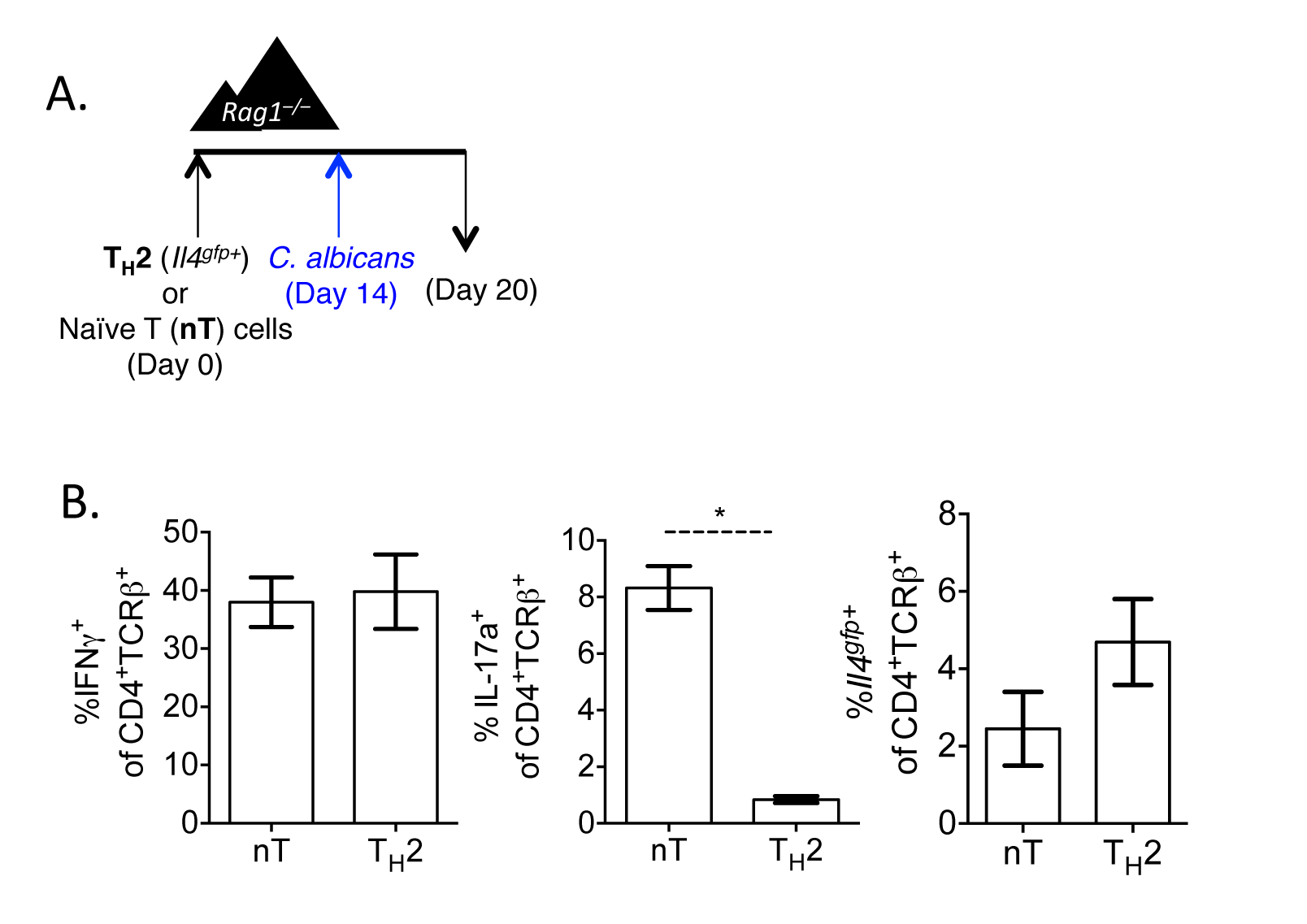

Supplement: S4 Fig — A). Experimental set-up: 2 week in vitro polarized Th2 cells were FACS sorted as CD4+ Il4 gfp+ and transferred i.v. to Rag1 –/–mice. As a control, a group of Rag1 –/–mice received naïve CD4+ T cells. Recipient mice were infected with 105 C. albicans yeast forms i.v. on day 14 post-transfer and harvested at day 6 post-infection. B). Percent of CD4+TCRβ+ cells producing IFNγ, IL-17a, or GFP (IL-4) in the spleen, as determined by intracellular cytokine staining. Data are representative of 4 separate experiments, with 3–5 mice per group. (TIF) [file ppat.1004994.s004.tif]

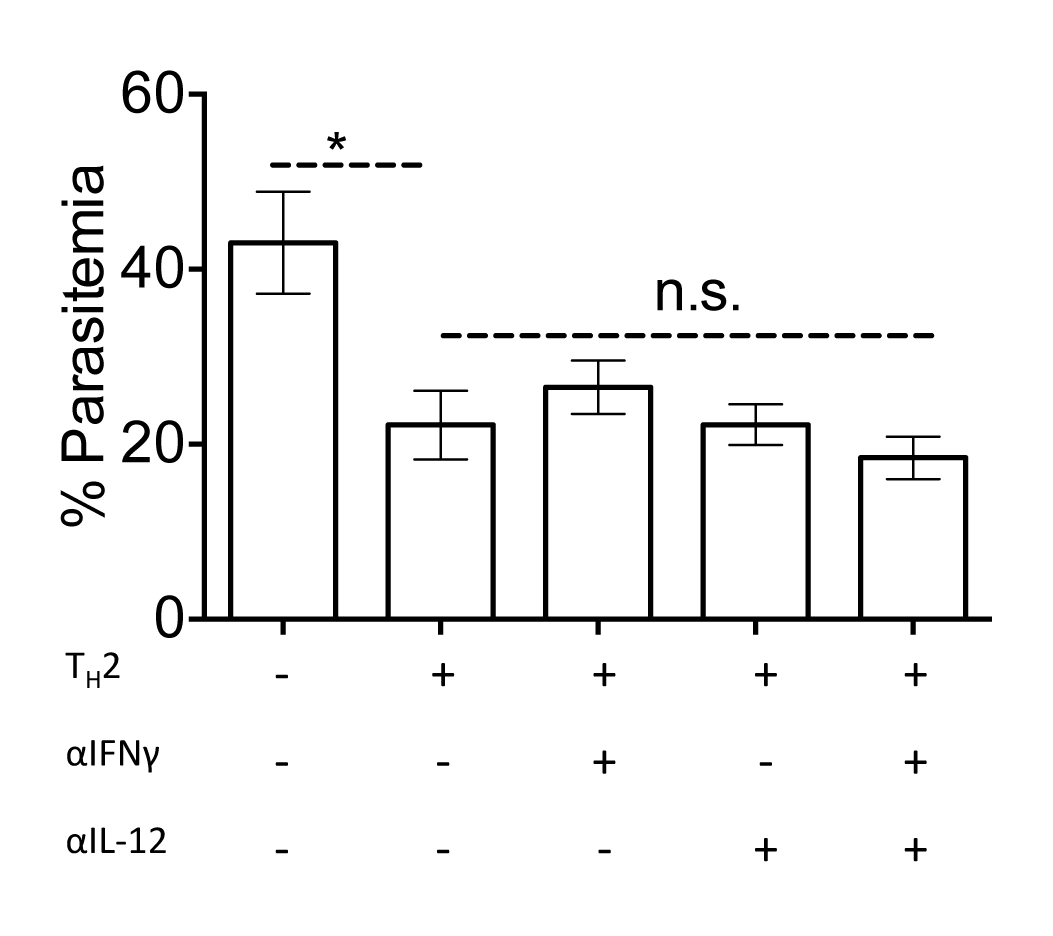

Supplement: S5 Fig — In vitro Th2 (CD4+TCRβ+ Il4 gfp+) cells were transferred to Rag1 –/–recipient mice for 14 days. Mice were infected with P. chabaudi and harvested at d8 post-infection. Mice were treated i.p. with 0.5mg anti-IL12 and anti-IFNγ at days -1, 6, 13, and 19, as shown in Fig 8A. Percent parasitemia was determined by blinded counting of Giemsa-stained blood smears. Data representative of 2 independent experiments with 3–5 mice per group. * denotes P<0.05. (TIF) [file ppat.1004994.s005.tif]

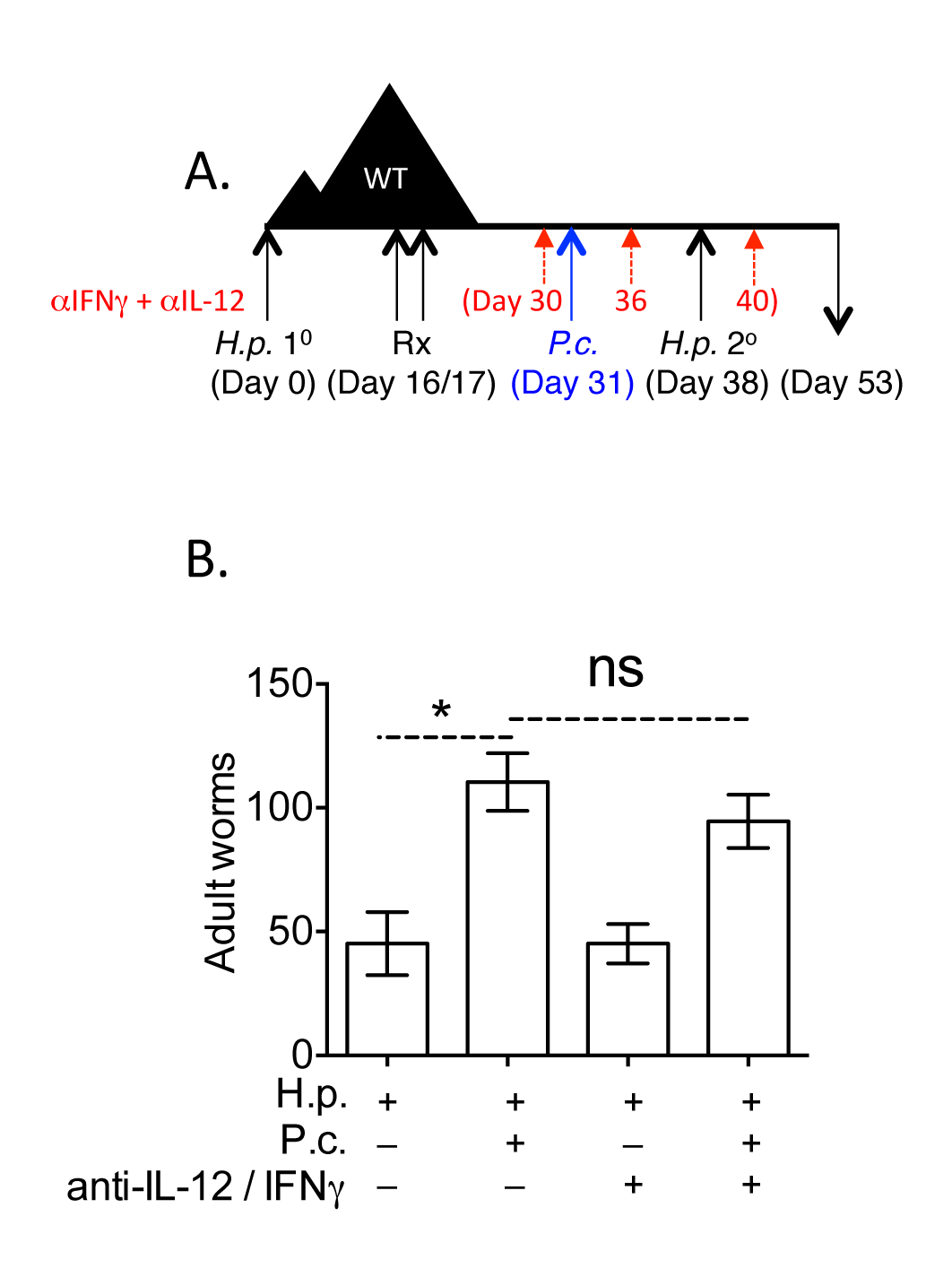

Supplement: S6 Fig — A). C57BL/6 mice were infected with 200 H. polygyrus larvae, treated on 2 consecutive days (days 16 and 17) with pyrantel embonate (5 mg), infected with 105 P. chabaudi (day 31) and re-infected with H. polygyrus (day 38). Mice were treated with 0.5 mg of anti-IL-12 and anti-IFNγ i.p. at days 30, 36 and 40. B). Adult worms in intestine were counted on day 53. Data are representative of 2 independent experiments with 5–7 mice per group. * denotes P<0.05. (TIF) [file ppat.1004994.s006.tif]
